# New evidence finds young people in Mainland China are now bicultural

**DOI:** 10.1111/bjop.12767

**Published:** 2025-01-13

**Authors:** Yi‐meng Wang, Feng‐yan Wang, Thomas Talhelm, Yi‐qun Chen

**Affiliations:** ^1^ School of Psychology Northwest Normal University Lanzhou China; ^2^ School of Psychology & Institute of Moral Education Research Nanjing Normal University Nanjing China; ^3^ Behavioral Science Booth School of Business, University of Chicago Chicago Illinois USA

**Keywords:** attribution, biculturalism, dialectical thinking, self‐concept, Taiji model of self

## Abstract

This study reports new evidence that young people in Mainland China are now bicultural. We followed the established method of testing biculturalism by priming participants with images from two different cultures and measuring whether those images activate different thought styles. First, we replicated findings from 25 years ago that college students in Hong Kong are bicultural (Study 1). Next, we found that priming Mainland Chinese college students with Chinese culture increased external attributions (which are more common in China), whereas priming American culture increased internal attributions (which are more common in the US; Study 2). Next, we tested a “negative control” group that we expected should *not* respond to bicultural primes. Older adults who were born before China's Reform and Opening policy in 1978 showed no evidence of biculturalism (Study 3). This new evidence extends biculturalism to Mainland China, and it provides a crucial negative control test for biculturalism research.

## BACKGROUND

In the early days of cultural psychology, most studies tried to test for differences between groups of people. Since 1997, researchers have found evidence of cultural differences *within the same people* (Hong et al., 1997, 2000). The idea is that some people are bicultural or even multicultural with three or more cultural identities (Hong & Schmidt, 2021). Multicultural people have different cultural identities, which have different scripts for how to think, what to value, and how to behave (LaFromboise et al., 1993; Phinney & Devich‐Navarro, 1997).

The contribution of this research was to demonstrate that cultural differences can be something other than stable trait differences between people. Instead, cultural differences can also include differences that become activated in the same people in different situations. This adds a layer of complexity to thinking of people as having *a* cultural identity. People can have multiple cultural identities.

## CULTURE AND BICULTURALISM

Researchers have defined culture as a set of socially transmitted information within a population that influences cognition, affect, and behaviour (e.g., Kashima, 2016). This information includes symbols, beliefs, values, norms, and practices shared among interconnected individuals (Lu et al., 2023). This system of culture is continuously reproduced through means such as language, media, and institutions, and it takes many forms, including national, ethnic, political, religious, technological, and social class cultures (Cohen & Varnum, 2016). While culture exists at a macro level—pertaining to communities, regions, and nations—individuals internalise these cultural values, allowing them to navigate multicultural environments and embody multiple cultural identities. Individuals can thus hold two or multiple cultural identities reflecting their belonging to different forms of culture (Hong et al., 2000; Morris et al., 2015).

Multicultural/bicultural individuals are people who have been exposed to and internalised two or more cultural systems (Benet‐Martínez, 2012; Hong et al., 2000). Agar (1991) was one of the first to explore the biculturalism of bilingual people, emphasising the distinctive cultural experiences and identities that arise from fluency in multiple languages. More recently, researchers have argued globalisation and immigration are making biculturalism increasingly common in many societies (Martin & Shao, 2016). These individuals are “fluent” in two cultures through close psychological or social engagement (Cheng et al., 2020), with each culture alternately influencing their thoughts and emotions (LaFromboise et al., 1993; Phinney & Devich‐Navarro, 1997). Consequently, biculturals adapt their behaviour to align with the norms and expectations of both cultures.

## PRIMING CULTURAL IDENTITIES

The psychological mechanism behind biculturalism follows the principles of knowledge accessibility, applicability, and salience. The core concept is that the pieces of an individual's knowledge vary in accessibility (Higgins, 1996; Hong et al., 2000). After people acquire cultural knowledge and internalise it, symbols of that culture can activate that knowledge, making it temporarily accessible (Hong et al., 2007). Bicultural people respond to those cultural symbols by switching between different cultural styles, thought processes, feelings, or behaviours in line with that cultural tradition (Chiu & Hong, 2018).

Researchers tested this idea by priming people in Hong Kong with symbols of Chinese versus Western culture. The idea was to see whether these primes would elicit behaviours and attitudes more like people in China versus the West. They chose Hong Kong because it is a meeting place between Eastern and Western culture (Hong et al., 1997). Hong Kong is majority Chinese, but it is a former British Colony. As a former colony, English is a common language of education. Hong Kong is also a hub of international trade, connecting products from China to buyers in the Western world.

They found that simply showing people pictures of different cultures was enough to change how they processed information in a way similar to studies of the US and China (Hong et al., 1997). This result suggested that at least some people in Hong Kong are bicultural, with both cultural mindsets ready to be activated. However, two unanswered questions remain: Are people in other societies also bicultural? Are there generational factors and changes in other psychological mechanisms associated with biculturalism? In this study, we test both of these questions.

Following Hong et al.'s (1997, 2000) studies, later researchers used the same cultural priming method to examine its effects on undergraduate students in Mainland China (Sui et al., 2006, Experiment 1). They found that participants in the Chinese cultural priming and control conditions endorsed interdependent self‐construal more than participants in the American priming condition.

Some researchers modified Hong and colleagues' cultural priming paradigm (Friedman et al., 2012; Martin & Shao, 2016). For example, Friedman et al. (2012) randomly assigned participants to either a Chinese or Western environmental priming condition. In the Chinese environmental priming, they hung three bamboo decorations on the walls. In the Western environmental priming, they hung seven Christmas cards. They found that this cultural priming method also influenced people's cultural styles.

We tried to stick closely to the priming method from the original study. We did this for two reasons. (1) We did this so we could test the effect across different regions and generations while keeping the methods similar to the original. (2) We wanted to replicate the original study as a part of some researchers' call for more replications (Open Science Collaboration, 2015). However, we should note that there are some slight differences in our design, so it is not a direct replication.

## CULTURAL DIFFERENCES IN CAUSAL ATTRIBUTION

Attributions are people's explanations for the causes of behaviour and events. A foundational premise of attribution theory is Heider's (1958) contention that perceivers seek to attribute fleeting behaviours to stable dispositions so that they can learn about the social environment.

Attributions can vary across cultures. Studies have found that people in East Asia tend to endorse external attributions more than people in Western cultures (review: Dean & Koenig, 2019; Morris & Peng, 1994; Spencer‐Rodgers et al., 2012). For example, people from China were more likely to attribute a murder to situational factors like stress or societal pressure than people from the US. In contrast, people in the US were more likely to endorse internal factors, such as the murderer's personality or mental illness (Morris & Peng, 1994).

In our study, we base our methods on a classic study of people's attributions for cartoon fish (Morris & Peng, 1994). Researchers showed students in China and the US animations of a blue fish swimming in a lake near a group of fish. In some scenes, the blue fish joined the group. In other scenes, the blue fish swam away from the group, or the group caught up to the blue fish. Next, the researchers asked participants to rate whether internal factors or external factors seemed to influence the fish's behaviour.

People in China tended to explain the fish's movement through external factors, whereas the American students tended to explain the fish's movement through internal factors. The researchers argued that these cultural differences in explanations are for social events, not for all events. This was evident in the fact that there were no cultural differences in how the students interpreted scenes of a circle and a rectangle.

In sum, the study found evidence of more external attributions for social events in China and more internal attributions for social events in the US. The cultural difference in this attribution seems to be fairly well founded. A recent replication study found differences similar to the original study (Cao et al., 2024).

Researchers used this basic finding to test whether they could prime people with bicultural identities (Hong et al., 2000). They showed college students in Hong Kong images that represented Chinese culture or Western culture. After seeing pictures of Chinese culture, they gave more external attributions than after seeing pictures of Western culture. This result suggested that cultural priming can temporarily influence people's cognitive style.

More recent studies have looked at what they call “innate multiculturals” and “achieved multiculturals” (Martin & Shao, 2016). Innate multiculturals are people who are raised in a multicultural household or immigrate abroad at a young age. For example, innate multiculturals would include a person with a Chinese mother and an Italian father living in Canada, or people with a Chinese cultural background who were born in Canada or immigrated there at a young age. Achieved multiculturals are people whose experience comes later and runs less deep, such as international students or people who immigrate at a later age.

When researchers tested these two groups of multiculturals, they found different results (Martin & Shao, 2016). Innate multiculturals attribution styles remained the same regardless of priming. The authors argued that this could be because the experience of early immersive culture mixing leads them to develop a single hybrid cultural cognition that guides their interpretations at all times, meaning that they do not switch between cultural frames. In contrast, achieved multiculturals' attribution became more external after Chinese cultural priming than Australian cultural priming. This is because the experience of engaging with multiple cultures sequentially or in different contexts leads them to develop distinct cultural cognitions. As a result, they may be more susceptible to cultural priming.

However, the bicultural studies in Hong Kong suggest that priming effects work with innate biculturals too—if people in Hong Kong count as innate biculturals. We suspect they do because the exposure to Western culture happens directly in Hong Kong and from a young age. For our samples in Mainland China, the vast majority of participants do not have a deep experience outside of China from a young age, so they would fit more closely with the achieved bicultural category.

Note that biculturalism research does not mean that monocultural people are unable to change how they process information. There are plenty of studies showing that people can change their social or cognitive style in response to different primes. For example, writing about the self makes people temporarily more interdependent than writing about relationships (such as Xi et al., 2018). It would be hard to believe that Americans are fundamentally incapable of giving external attributions or thinking about how relationships with other people shape who they are. Instead, the claim is that only bicultural people will respond to symbols of a culture by changing their thought style or social style to fit with the pattern of that culture.

### Extension: is Mainland China now bicultural?

If we can reproduce the original Hong Kong findings, that would suggest that our methods are stable compared to the earlier study (Hong et al., 2000). If so, that would set us up to be able to test whether the phenomenon of bicultural identity now extends to Mainland China.

The societal conditions in Hong Kong made it a logical place to do the first bicultural study. In the 1990s, Mainland China was far less open and less connected to the outside world than Hong Kong. This made Hong Kong an ideal place to test the theory of bicultural identities.

But since the 1990s, much has changed in Mainland China. People born after the 1990s have had unprecedented opportunities to interact with people from different cultural backgrounds (Economist, 2020). Take Chinese students studying in the US as an example. Based on statistics from the Institute of International Education, there was a negligible number of Mainland Chinese students in the US in 1980 (Economist, 2020; IIE, 2020). By 2019, there were over 300,000 Mainland Chinese students in the US.

Studying abroad is just one example of China's new openness. Compared to the 1970s, Mainland China now sees more Western tourists, more Western media, and more Western brands, such as Starbucks, Kentucky Fried Chicken, and Apple. This process of opening was kickstarted in 1978 with China's Reform and Opening policy, which let in foreign investment and foreign companies. In 1978, China received 230,000 international tourists (Lew, 1987). In 2019, China received 162 million (World Bank, 2020). In short, Mainland China has become far more connected to the outside world.

Mainland China's increasing openness raises the question of whether some people in Mainland China are now bicultural. Has the increasing exposure to Western now made Western ways of thinking an accessible part of people's identities? We look into this question by running Hong and colleagues' test of bicultural identity among Mainland Chinese college students.

### Extension: is biculturalism in China generational?

We also extend previous research by asking whether biculturalism in China falls along the historical opening of Chinese society. If young people in China are now bicultural, how do we know that this is a generational difference? It could be that people in China have long been bicultural.

After all, even when Chinese society was more closed after World War II, China was never completely closed. China's embrace of socialism brought in Western philosophical and political influences like Karl Marx. Even during China's most closed‐off period of the 20th century, Chinese dancers performed the Western ballet Swan Lake to large crowds (Duchen, 2011). If we find that young Chinese people are bicultural, we cannot just assume that older generations were not bicultural.

Thus, we tested for generational differences by comparing Chinese college students with older Chinese adults. Chinese college students grew up in a more open China, mostly after the year 2000. In contrast, our sample of older Chinese adults were born before China's Reform and Opening in 1978. Thus, they experienced a more closed period of Chinese history, particularly during their childhood, which is a critical period for identity formation (Schmitt‐Rodermund & Vondracek, 1999).

This generation serves as a “negative control” group for comparison with the younger generation. Negative controls are crucial in scientific experiments. The entire theory of biculturalism is that people need to have experience or broad exposure to another culture in order to respond to primes of that culture. Thus, we need to test people without exposure in order to test the theory that it is the exposure that is crucial for the effect. Yet all the studies we could find only tested people the researchers thought were bicultural.

Negative controls are important because it is possible that cultural priming would have the same effect even for people who know nothing about that culture. For example, maybe there are basic features in the images that influence people's thought styles. This would be consistent with a study that found that city landscapes were more “busy” in Japan, with more distinct objects and more overlap than in the US (Miyamoto et al., 2006). Simply showing people these city landscape pictures temporarily shifted their cognitive styles.

Our study offers a crucial test of the idea that people need cultural exposure to show biculturalism effects. Testing older participants in China is also a critical test for our explanation of cultural change in China over time.

### Dialectical self‐concept

In this study, our main outcome measure is attribution style. However, we also test whether cultural priming influences people's dialectical self‐concept. People with dialectical self‐concepts are more likely to see the self as changeable (flexibility and adaptability), tolerant of contradiction (accommodating opposites), and holistic (connected with other people, material objects, and the metaphysical realm).

Researchers have argued that people in East Asia's self‐concepts are more dialectical than people in the West (Nisbett et al., 2001; Peng & Nisbett, 1999; Spencer‐Rodgers et al., 2009). For example, people in Japan and China more readily brought to mind contradictory self‐knowledge than European Americans (Spencer‐Rodgers et al., 2009). People in Japan and China also said they were more likely to change over time, and they showed more inconsistency in their implicit self‐beliefs than European Americans.

Thus, we tested whether cultural priming would influence people's dialectical self‐concept. We tested dialectical self‐concept because (1) studies have found East–West cultural differences in dialectical self‐concept (Spencer‐Rodgers et al., 2009); (2) a recent study found that asking Chinese international students to write about their experience abroad made them more likely to endorse dialectical statements than asking them to think about their hometown (Hu et al., 2021).

This study abroad finding raises an intriguing question. Many Chinese students study abroad in Western cultures, where people endorse dialectical thought less (Peng & Nisbett, 1999). Thus, it seems paradoxical that priming study abroad would increase dialectical thinking. Instead, it seems like priming East Asian culture should increase dialectical thinking. However, priming a foreign culture could make people think about the contradictions between their home identity and the foreign culture, which could highlight how dialectical their identity is.

## STUDY OVERVIEW

It has been 25 years since the pioneering biculturalism study in Hong Kong, and a lot has changed since then (Hong et al., 1997). In the year of the first study, the UK returned Hong Kong to China. Hong Kong has become more politically and culturally connected to Mainland China (Wong, 2022). From 1996 to 2016, the number of Hong Kongers speaking Mandarin Chinese at home rather than the local Cantonese dialect doubled (Kao, 2018). This change makes it worth asking whether people in Hong Kong are as bicultural as they were in the past.

In Study 1, we replicated the original bicultural self‐study in Hong Kong, with one methodological improvement and one difference from the original study. We made slight changes to the design so that we could separate the internal and external attribution questions. This allowed us to test whether separating the internal and external attribution questions would change the results.

Additionally, we used the fish images from Morris and Peng's original animation to try to recreate the earliest research on biculturalism (Morris & Peng, 1994; Hong et al., 1997, Experiment 2). This is slightly different from the study of Hong et al.'s (2000) later study, which used a realistic picture of a fish swimming. Modifying the animations allows us to test whether the earlier results generalise across different versions of an attribution task. In Study 2, we tested whether students in Mainland China are now bicultural. In Study 3, we tested a sample of older Chinese adults to see if biculturalism is generational.

## TRANSPARENCY AND OPENNESS

All data, analysis code, and research materials are available at https://osf.io/u26zh/?view_only=6414baed370d4f91a93f2a737ba4961e. The study design and analysis were not preregistered.

## STUDY 1

In Study 1, we replicated the study of Hong et al. (1997), Experiment 2, and the “initial test” from Hong et al., 2000. We tested whether people in Hong Kong are still bicultural, which would replicate the findings from Hong et al. (1997, 2000).

## METHODS

### Participants

After receiving review and approval from the Chinese University of Hong Kong, we invited a postdoctoral researcher to send a mass email to students. The email contained a link to an informed consent form, instructions for participation, a Tencent questionnaire (wj.qq.com), and details on compensation. Tencent Questionnaire is a Chinese crowdsourcing platform similar to MTurk. The original questionnaires, data, and analysis scripts are available on the OSF. The study was not pre‐registered.

We included an attention check question: “Please choose 5 for this question” (Huang et al., 2012). We excluded 4 participants who failed the attention check. We also excluded 9 participants whose response times were more than 3 standard deviations from the mean. However, the results were similar if all participants were included (Data S1).

Our final sample consisted of 107 participants. All of them were students (35.5% male, 93.3% undergraduate students, 6.54% associate degree students or lower, and 14.02% master's students or higher; *M*_age_ = 21.41, *SD*_age_ = 3.26; age range = 18–34; 5.6% of participants had studied abroad in Western countries, such as America, Canada, and Australia). A sensitivity analysis using G*power found that a sample size of 107 has at least 80% power to detect an effect size of *f* = 0.30 in a between‐factor ANOVA (China, US, Control). This effect size of *f* = 0.30 is slightly smaller than that of Hong et al.'s, 2000 study (*N* = 75, *f* = 0.37) but larger than that of Hong et al.'s, 1997 study (*N* = 75, *f* = 0.18). Table S1 reports descriptive statistics for all demographics. Participants gave informed consent and received payment for participating.

### Procedure

This study had three phases: (1) cultural priming, (2) attribution task, and (3) Dialectical Self Scale (DSS). We randomly assigned participants to see primes of American culture (*N* = 36), Chinese culture (*N* = 35), or neutral pictures (*N* = 36). Then, in an ostensibly unrelated task, participants took measures of attribution and dialectical self‐concept.

### Cultural primes

**FIGURE 1:**
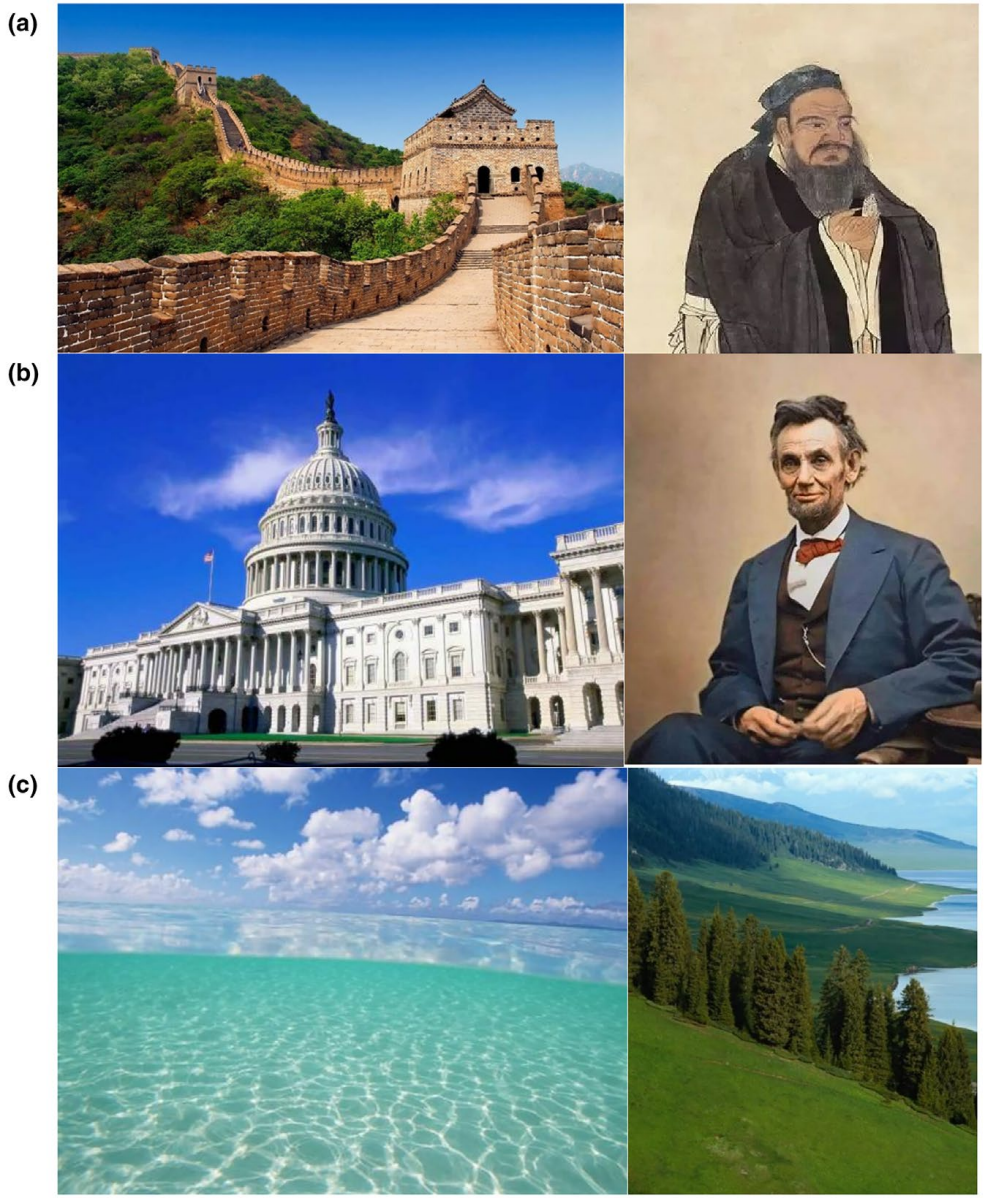
Examples of Iconic Images in (a) Chinese Culture, (b) American Culture, and (c) Landscapes. These cultural priming images come from a study by Li (2020).

We used pictures of cultural icons (Figure 1) to prime different cultural identities from a previous study (Li, 2020). We used symbols (such as the American flag and a Chinese dragon), food items (such as knives and forks vs. chopsticks), famous people (such as Abraham Lincoln vs. Confucius), and landmarks (such as the US Capitol vs. the Great Wall).

We wanted to use the same pictures as the original study by Hong et al. (2000), but the paper only included a few example pictures. We found priming materials on the author's website, but some images described in the original paper were missing from the materials (such as Superman, the US Capitol, and the Monkey King). Thus, we supplemented the images that the original paper included with additional pictures from a later study of cultural priming (Li, 2020).

Prior studies have found that seeing cultural icons primes people with different cultural meaning systems. For instance, Hong et al. (2000) found that exposing Hong Kong Chinese students to these Chinese icons increased their endorsement of Chinese values. Kemmelmeier and Winter (1998) found that showing Americans the American flag increased their endorsement of independence values.

Participants in the American culture priming condition saw eight pictures of American icons. Participants in the Chinese culture priming condition saw eight pictures of Chinese icons. These two groups answered two questions about each picture: “How much do you like this picture?” from 1 (*strongly dislike*) to 10 (*strongly like*) and “Which country does this picture symbolize?” Participants in the control condition saw eight pictures of nature and the liking questions, but not the questions about countries.

### Attribution task

**FIGURE 2:**
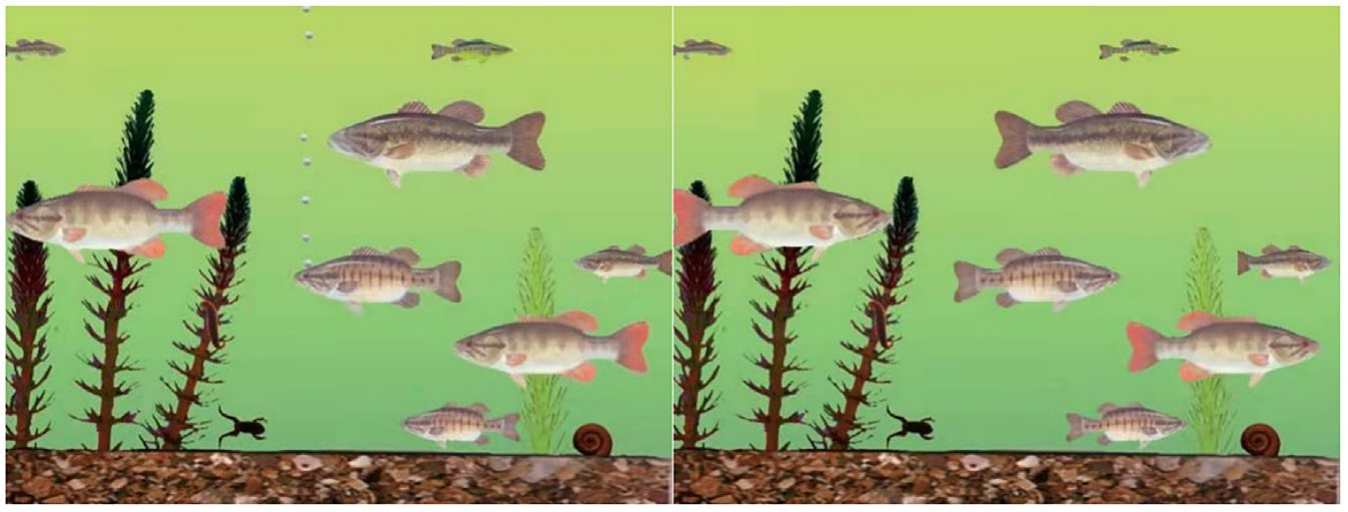
Fish Animations Used to Measure Internal and External Attributions. These images are adapted From Nisbett et al. (2001).

To measure attribution style, we used the “fish task” from previous research (Hong et al., 1997; Masuda & Nisbett, 2001). Participants saw two cartoon images (in a random order) of a fish swimming in front of a group of fish (Figure 2, left) and behind a group of fish (Figure 2, right). After seeing the images, participants rated explanations for why the fish was swimming in front of and behind the other fish.

We calculated scores as internal attributions, averaging over five questions for each of the two cartoons. We reverse‐scored the external attribution questions. For example, one item asked, “Why do you think there is one fish swimming in front of/behind the group of fish?” Participants responded from 1 (*very confident that it is because the one fish is leading/independent from the other fish* [an internal attribution]) to 5 (*very confident that it is because the one fish is being chased/rejected by the other fish* [an external attribution]). We also tested whether separating these items would make a difference because it is not clear whether (1) internal and external attributions are opposite ends of a single continuum or (2) internal and external attributions are separate, such that some people endorse both at the same time. Data S1 lists all attribution wordings.

### Dialectical self‐concept

Finally, participants completed a brief version of the 14‐item Dialectical Self Scale (DSS; Spencer‐Rodgers et al., 2016). This scale measures whether people think of the self in a dialectical way, such as being sensitive to different contexts and changing often (Spencer‐Rodgers et al., 2016). Participants rated agreement with statements like “I often change the way I am, depending on who I am with” from 1 (*strongly disagree*) to 7 (*strongly agree*). The reliability (Cronbach's α) for this scale was .56.

Because the scale showed low reliability, we improved the scale by removing items with poor item‐total correlations. For example, several items had negative item‐total correlations, which should not happen. We are also correcting for acquiescent response style. Acquiescence is a commonly observed response style that may distort respondent scores. Thus, we controlled for acquiescence by subtracting participants' average agreement (balanced across negative and positive items) from their response to each item (Primi et al., 2019; the Data S1 report more details on response style). After correcting for acquiescence and removing items with poor item‐total correlations, the scale had 9 items and a reliability of .64, which is above the commonly used cutoff of .60 (Cohen et al., 2017). We also tested the uncorrected scores of the Dialectical Self‐Concept Scale, and the results were consistent with those of the corrected scale (Data S1).

## RESULTS

### Descriptive analyses

Table 1 reports means, standard deviations, and correlations between the variables. Demographics had no significant relationships with attributions or dialectical self‐concept.

**TABLE 1:** Correlations and descriptive statistics for study 1.

	*M*	*SD*	1	2	3	4	5	6
1. Gender	1.64	0.48	–					
2. Age	21.41	3.26	−.07	–				
3. Education	2.09	0.51	−.17	.51***	–			
4. Experience Abroad (%)	14.95%	0.36	.09	.14	−.08	–		
5. Liking for Pictures	6.64	1.43	−.01	.18	.14	.07	–	
6. Internal Attributions (Combined)	3.00	0.42	−.01	−.10	−.01	−.14	−.10	
7. Dialectical Self‐Concept	4.28	0.52	.18	−.19	−.17	.06	−.08	−.02

*Note*: Gender is coded 1 = male, 2 = female. Experience abroad is coded 0 = no, 1 = yes. Education is coded 1 = less than an associate degree or equivalent, 2 = bachelor's degree, 3 = master's degree or higher. Liking is coded from 1 (*strongly dislike*) to 10 (*strongly like*).

***p < .001.

### Attributions

**FIGURE 3:**
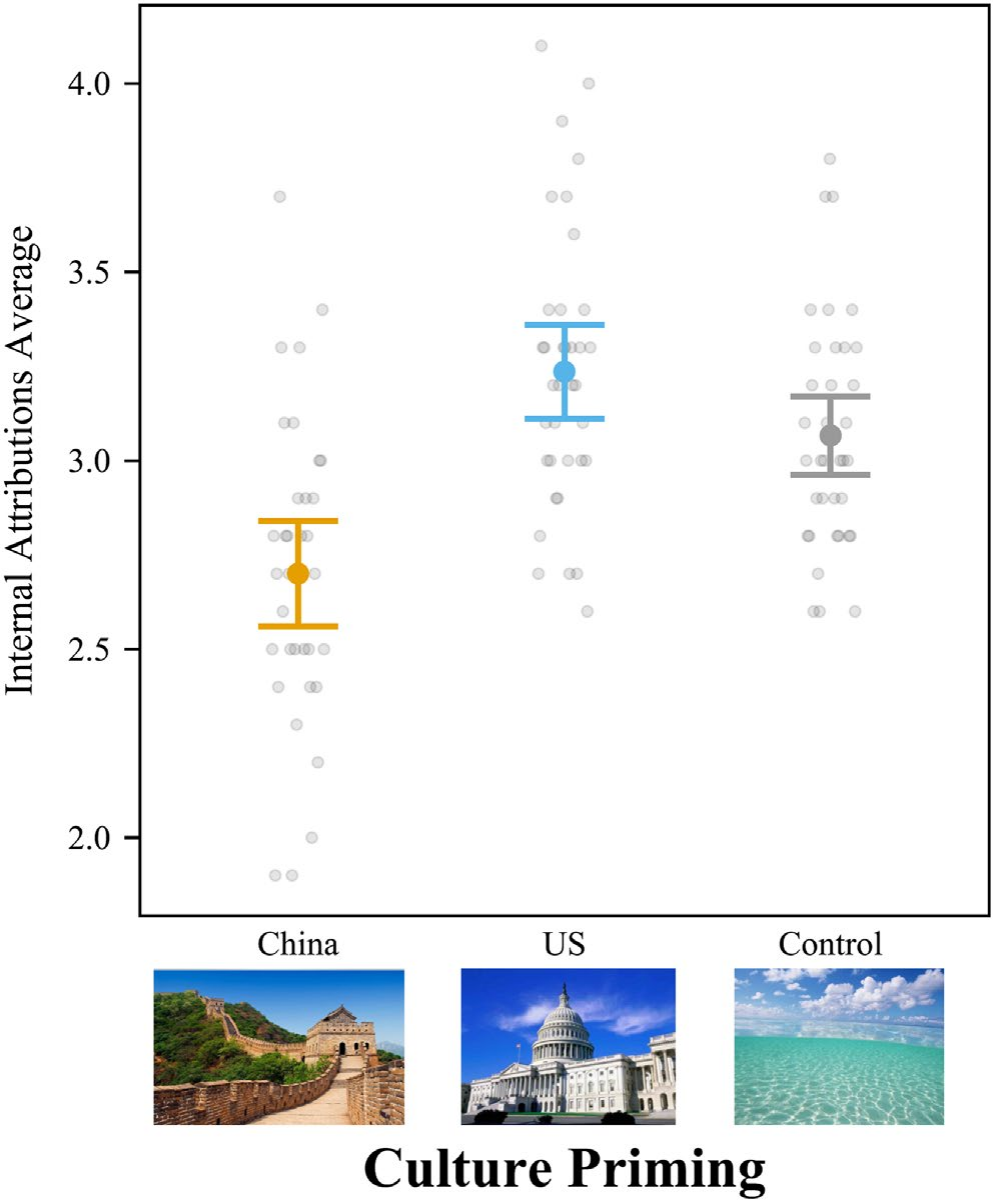
Priming American Culture Increased Internal Attributions for Hong Kong College Students. The internal attribution score is calculated as the average of 10 questions. Participants rated the internal attribution from 1 to 5. Bars = 95% confidence intervals. Each dot represents a participant.

We conducted a one‐way ANOVA on attributions across the three conditions (1 = Chinese prime, 2 = American prime, 3 = neutral prime) and found that cultural priming significantly affected internal attributions, *F*(2,104) = 20.23, *p* < .001, *f* ^*2*^ = .388 [.190, .626] (Figure 3). Post‐hoc Tukey HSD tests showed participants primed with American culture agreed more with internal attributions (*M* = 3.24; *SD* = 0.37) than participants primed with Chinese culture (*M* = 2.70; *SD* = 0.41), *t*(104) = 6.23, *p* < .001, *d* = 1.479, 95% CI = [0.915, 2.044]. People in the control condition (*M* = 3.07; *SD* = 0.31) also gave more internal attributions than participants primed with Chinese culture, *t*(104) = 4.26, *p <* .001, *d* = 1.012, 95% CI = [0.447, 1.576]. Finally, participants in the American culture condition gave more internal attributions than participants in the control prime condition, but it was right at the cutoff for statistical significance, *t*(104) = 1.98, *p* = .121, *d* = 0.467, 95% CI = [−0.093, 1.028]. The internal attribution pattern replicated the findings of Hong et al. (1997, 2000).

We also ran ANOVAs controlling for all demographic variables. As before, the results showed that culture priming significantly influenced internal attributions, *F*[2,99] = 18.33, *p <* .001, *f* ^2^ = .370 [.174, .608].

### Dialectical self‐concept

**FIGURE 4:**
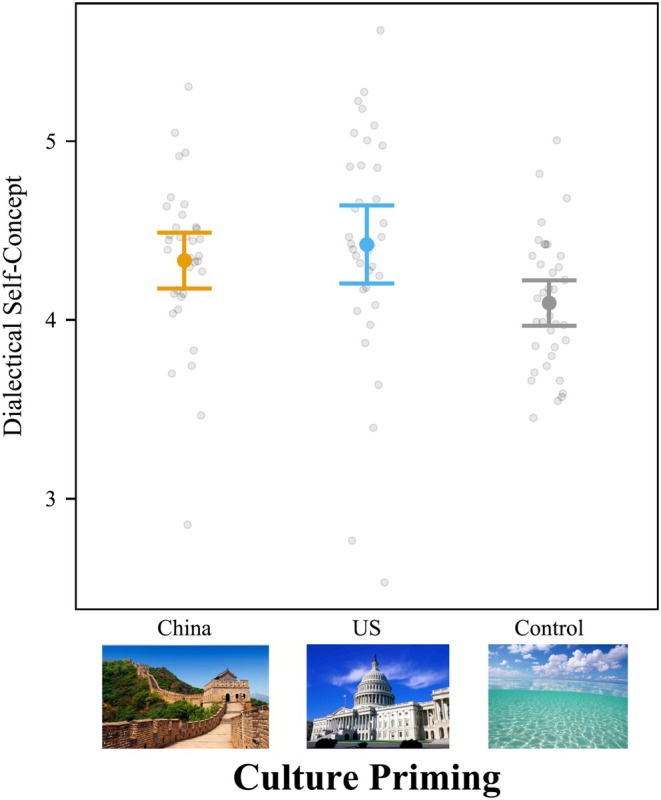
American Culture Priming Increased Dialectical Self‐Concept for Hong Kong College Students. Participants rated 14 items on self‐concept from 1 (*strongly disagree*) to 7 (*strongly agree*), such as “I sometimes believe two things that contradict each other.” Bars = 95% confidence intervals. Each dot represents a participant.

Culture priming had a significant effect on dialectical self‐concept without control variables, *F*(2,104) = 4.06, *p =* .020, *f* ^*2*^ = .078 [.007, .184]. Controlling for all demographics, the main effect of culture priming on dialectical self‐concept was not significant, *F*(2,99) = 2.29, *p =* .107, *f* ^2^ = .046 [.000, .133] (Figure 4). Surprisingly, post‐hoc Tukey HSD tests showed priming with American culture increased dialectical self‐concept ratings (*M* = 4.42; *SD* = 0.65) over the neutral priming (*M* = 4.09; *SD* = 0.37, *t*[104] = 2.76, *p =* .019, *d* = 0.650, 95% CI = [0.090, 1.211]). The difference between the American and Chinese priming was not significant (*M* = 4.33; *SD* = 0.45, *t*[104] = 0.75, *p =* .734, *d* = 0.178, 95% CI = [−0.386, 0.743]). The difference between the Chinese and neutral priming was also not significant (*t*[104] = 1.99, *p =* .120, *d* = 0.472, 95% CI = [−1.037, 0.092]).

This result is at odds with the idea that the dialectical self is more common in East Asia (Spencer‐Rodgers et al., 2009). However, one possible explanation is that reminding Hong Kong college students that they also have a Western identity may also remind them that they have conflicting identities, which highlights the dialecticism of the self. At the same time, reminding Hong Kong college students of Chinese culture may prime dialectical thought in a way consistent with typical cultural priming effects.

## STUDY 2

In Study 2, we extended Study 1 to Mainland China. Mainland China offers an interesting test case because it is less obviously bicultural than Hong Kong. However, as China has modernised and opened up to the outside world, people may have more bicultural experiences than before. For example, youngsters may like eating McDonald's and watching Transformers movies but also believe in traditional Chinese medicine and filial piety.

To test the possibility that biculturalism is on the rise after China opened up, we recruited college students to represent the younger generation. Starting in 1978 and accelerating through the 2000s, China opened up to more foreign trade, foreign companies, travel, and study exchanges. Thus, the generation born after the Reform and Opening period should, on average, have grown up in a more bicultural era of Chinese history.

## METHODS

### Participants

We recruited 300 college students from Tencent Questionnaire (wj.qq.com). We used the same attention check question as in Study 1 (“Please choose 5 for this question”). We excluded 28 participants because they failed the attention checks (*n* = 10) or because they were not college students (*n* = 16). In Data S1, we present analyses without the exclusion criteria.

Thus, the final sample for Study 2 included 272 college students (37.7% male, 97.1% undergrad or higher, *M*_age_ = 22.37, *SD*_age_ = 3.85; age range = 17–38, 8.5% of participants had studied abroad). A sensitivity analysis using G*power found that a sample size of 272 has at least 80% power to detect an effect size of *f* = 0.19 in a between‐factor ANOVA (China, US, Control). Table S1 reports full descriptive statistics. Participants gave informed consent and received compensation for completing the study.

### Procedure

We randomly assigned the participants to one of the three cultural primes (China, *N* = 95; America, *N* = 83; landscape, *N* = 94). We then tested attribution style and dialectical self‐concept. As in Study 1, we used the refined 9‐item Dialectical Self‐Scale; the reliability was *α* = .54 for college students. The reliability for college students was above a recommended cutoff of .50 but below a more common cutoff of .60 (Cohen et al., 2017; Hinton et al., 2014). The low reliability is a limitation of Study 2. We also tested the uncorrected scores of the Dialectical Self‐Concept Scale (Data S1).

## RESULTS

### Descriptive analyses

Table 2 reports the means, standard deviations, and correlations. Liking ratings of pictures and experience studying abroad were significantly correlated with internal attributions among college students. Demographic differences had no significant relationships with dialectical self‐concept. Attributions were not significantly correlated with dialectical self‐concept.

**TABLE 2:** Descriptive statistics and correlations in study 2.

	*M*	*SD*	1	2	3	4	5	6
1. Gender	1.63	0.48	–					
2. Age	22.37	3.85	−.02	–				
3. Education	1.38	0.54	.02	.19***	–			
4. Experience Abroad	8.46%	0.28	.01	.25***	.40***	–		
5. Liking of Pictures	6.48	2.18	−.03	−.00	.02	−.06	–	
6. Internal Attributions (Combined)	3.16	0.38	.00	−.04	−.01	−.22***	−.27***	–
7. Dialectical Self‐Concept	4.69	0.50	−.02	.02	−.01	.01	.00	.02

*Note*: Gender is coded 1 = male, 2 = female. Experience abroad is coded 0 = no, 1 = yes. Education is coded 1 = less than high school and equivalent, 2 = vocational degree, 3 = bachelor's degree, 4 = master's degree or higher. Participants rated liking from 1 (*strongly dislike*) to 10 (*strongly like*).

***p < .001.

### Attributions

**FIGURE 5:**
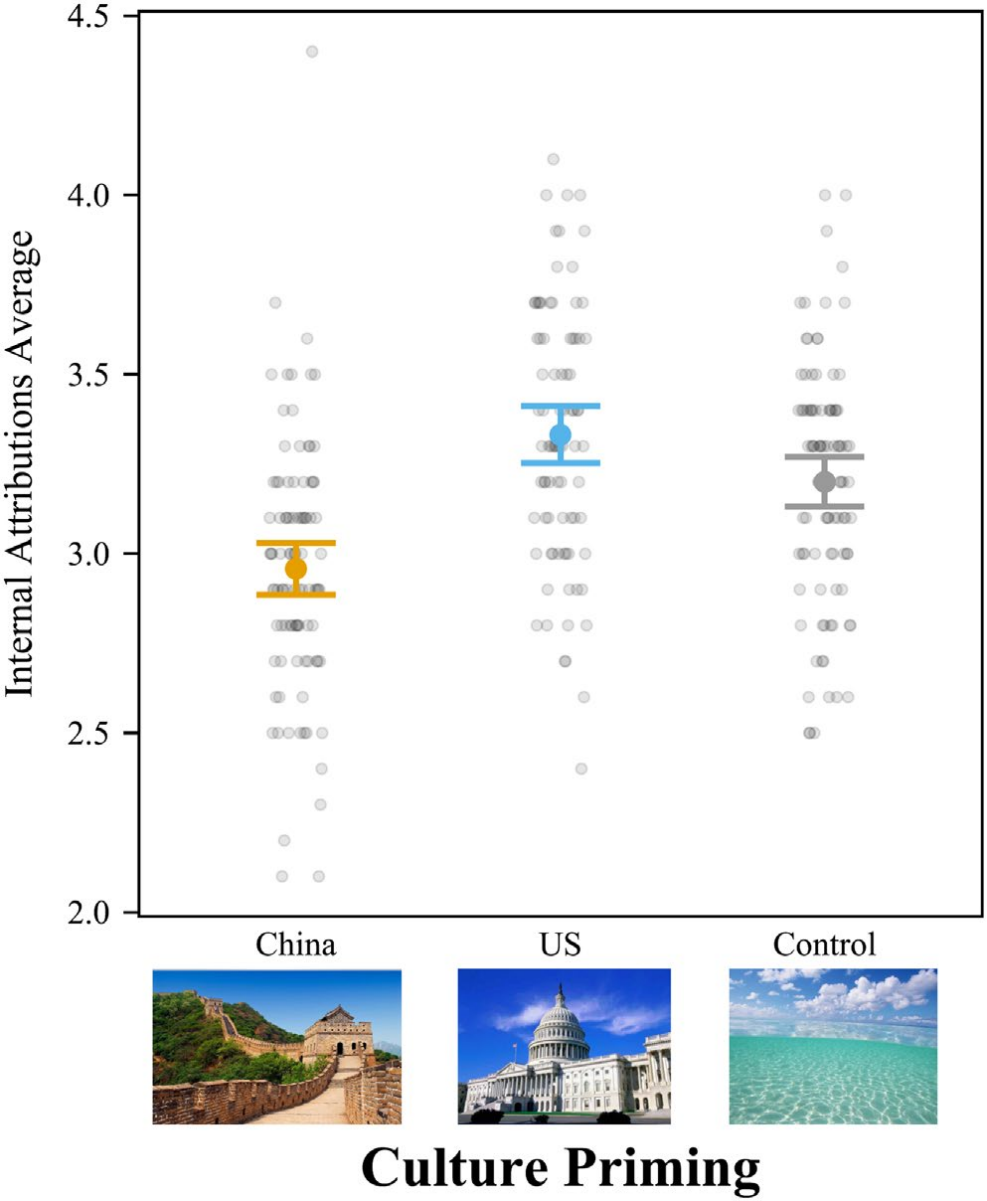
Priming American Culture Increased Internal Attributions for College Students in Mainland China. Participants rated 10 attribution questions from 1 to 5. Bars = 95% confidence intervals. Each dot represents a participant.

Cultural priming significantly affected internal attributions, *F*(2, 269) = 26.15, *p <* .001, *f* ^*2*^ = .195 [.110, .294] (Figure 5). We also ran ANOVAs controlling for all demographic variables, and the results were similar. Culture priming significantly influenced internal, *F*(2,264) = 13.19, *p <* .001, *f* ^2^ = .100 [.042, .172].

Students primed with American culture gave more internal attributions (*M* = 3.33; *SD* = 0.36) than students primed with Chinese culture (*M* = 2.96; *SD* = 0.35), *t*(269) = 7.07, *p <* .001, *d* = 1.062, 95% CI [0.708, 1.417], and participants in the control condition (*M* = 3.20; *SD* = 0.34), *t*(269) = 2.48, *p =* .036, *d* = 0.374, 95% CI [0.019, 0.729]. Additionally, participants in the control condition gave more internal attributions than students primed with Chinese culture, *t*(269) = 4.74, *p <* .001, *d* = 0.689, 95% CI [0.346, 1.032].

In sum, students primed with Chinese culture attributed less to the internal disposition of the fish than students primed with American culture. The results for the control condition fell in between the Chinese and American priming conditions. These findings suggest that college students in Mainland China are bicultural.

### Dialectical self‐concept

**FIGURE 6:**
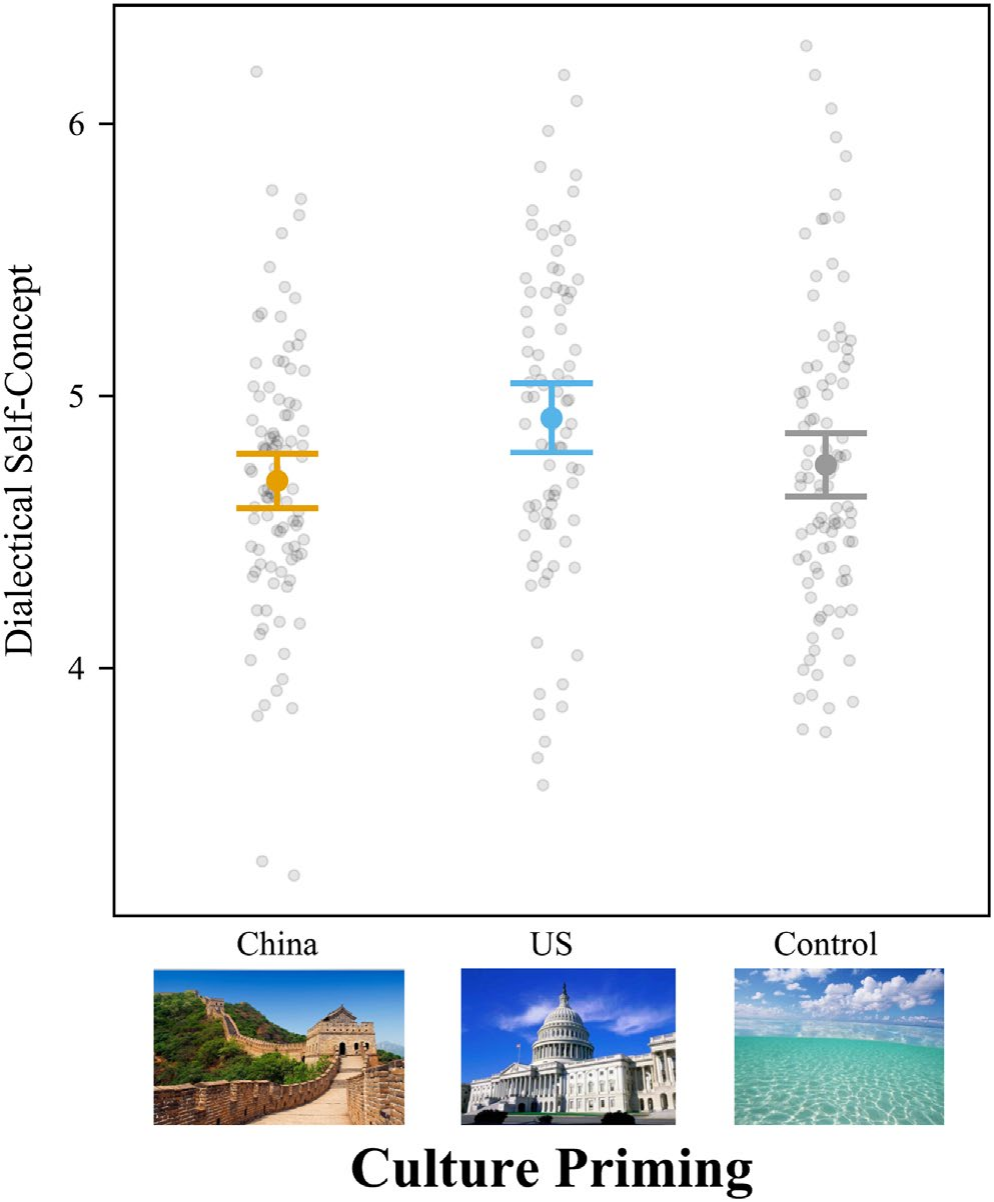
Priming American Culture Increased Dialectical Self‐Concept for College Students in Mainland China. Participants rated self‐concept questions from 1 (*strongly disagree*) to 7 (*strongly agree*), such as “I sometimes believe two things that contradict each other.” Bars = 95% confidence intervals. Each dot represents a participant.

Cultural priming significantly influenced college students' dialectical self‐concept, *F*(2,269) = 4.19, *p =* .016, *f* ^*2*^ = .031 [.003, .072] (Figure 6). The results were similar when controlling for all demographic variables. Culture priming significantly influenced dialectical self‐concept, *F*(2,264) = 7.19, *p* < .001, *f* ^2^ = .055 [.014, .109]. Priming college students with American pictures led to higher ratings of dialectical self‐concept (*M* = 4.92; *SD* = 0.58) than priming with Chinese pictures (*M* = 4.69; *SD* = 0.50), *t*(269) = 2.81, *p* = .015, *d* = 0.422, 95% CI [0.068, 0.776]. Like Study 1, American cultural priming also led to a higher dialectical self‐concept than neutral priming, but it was marginally significant, *M* = 4.75; *SD* = 0.57, *t*(269) = 2.09, *p* = .095, *d* = 0.314, 95% CI [−0.041, 0.669]. The difference between Chinese priming and the neutral priming was not significant, *t*(269) = 0.74, *p* = .739, *d* = 0.108, 95% CI [−0.235, 0.451].

## DISCUSSION

Studies 1 and 2 replicated the finding that college students in Mainland China are bicultural, similar to earlier studies of college students in Hong Kong (Hong et al., 2000). One surprising finding was that priming American culture caused students to rate their self‐concept as more dialectical. We had expected that priming people with Chinese culture would cause a more dialectical self‐concept. One explanation is that priming American culture reminds Chinese students of their conflicting identities, which makes them think of themselves as more dialectical. In contrast, priming their Chinese identity does not highlight their conflicting identities.

## STUDY 3

In Study 3, we recruited older people born before China's Reform and Opening in 1978 to represent a less globalised generation. Compared to the younger generation, the generation born before China's Reform and Opening experienced less foreign trade, foreign companies, travel, and study exchanges. Thus, they should have grown up in a less bicultural era of Chinese history on average.

## METHODS

### Participants

We recruited 300 older adults from Tencent Questionnaire (wj.qq.com). We used the same attention check question as in Studies 1 and 2 (“Please choose 5 for this question”). We excluded 42 older adults because they failed the attention check question (*n* = 9) or their age did not fit our criteria (*n* = 33). In Data S1, we present analyses without exclusion criteria.

Thus, the final sample for Study 3 included 258 older adults (54.3% male, 59.7% with an undergrad degree or higher, *M*_age_ = 55.70, *SD*_age_ = 5.18; age range = 43–70, 5.4% of participants had studied abroad). A sensitivity analysis using G*power suggested that a sample size of 258 has at least 80% power to detect an effect size of *f* = 0.19 in a between‐factor ANOVA (China, US, Control). Table S1 reports full descriptive statistics. Participants gave informed consent and received compensation for completing the study.

### Procedure

We randomly assigned the participants to one of the three cultural primes (China, *N* = 78; America, *N* = 68; landscape, *N* = 112). We then tested attribution style and dialectical self‐concept. The reliability of the 9‐item Dialectical Self‐Scale was *α* = .52. We also tested the uncorrected scores of the Dialectical Self‐Concept Scale (Data S1).

## RESULTS

### Descriptive analyses

Table 3 reports the means, standard deviations, and correlations. Demographic variables were not significantly correlated with internal attributions, external attributions, or dialectical self‐concept. Similar to Studies 1 and 2, attributions were not significant correlated with dialectical self‐concept.

**TABLE 3:** Descriptive statistics and correlations in study 3.

	*M*	*SD*	1	2	3	4	5	6
1. Gender	1.46	0.50	–					
2. Age	55.70	5.18	.03	–				
3. Education	2.65	1.25	−.15**	−.29***	–			
4. Experience Abroad	5.00%	0.23	.02	−.13*	.15**	–		
5. Liking of Pictures	7.74	1.82	−.04	−.02	.06	.01	–	
6. Internal Attributions (Combined)	2.94	0.44	−.01	.05	.06	.08	−.04	–
7. Dialectical Self‐Concept	3.98	0.67	−.01	.02	−.07	.00	−.07	.07

*Note*: Gender is coded 1 = male, 2 = female. Experience abroad is coded 0 = no, 1 = yes. Education is coded 1 = less than high school and equivalent, 2 = vocational degree, 3 = bachelor's degree, 4 = master's degree or higher. Participants rated liking from 1 (*strongly dislike*) to 10 (*strongly like*).

***p < .001.

**p < .01.

*p < .05.

### Attribution

**FIGURE 7:**
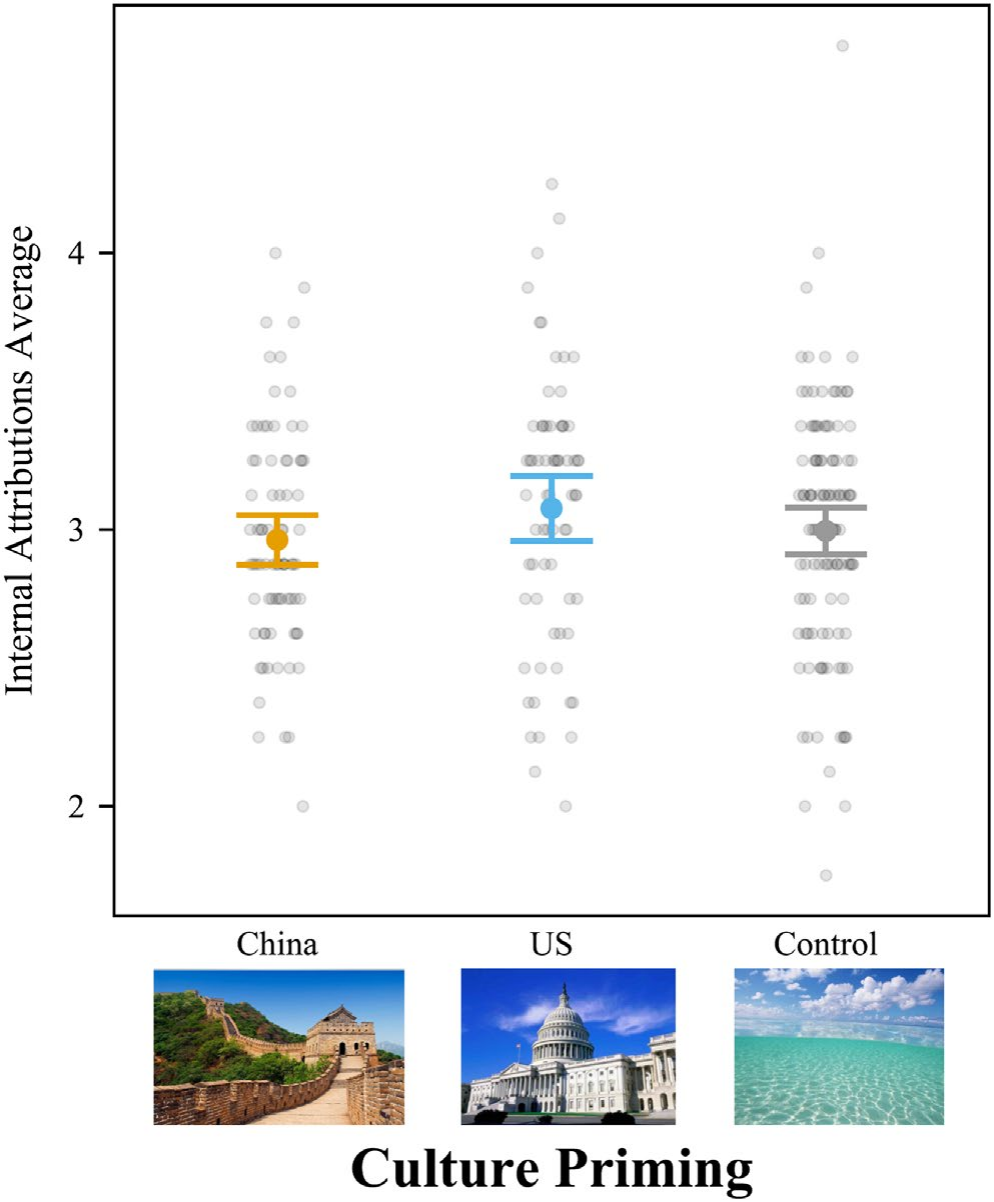
Cultural Priming Had No Effect on Attributions for Older Adults in Mainland China. Participants rated five attributions from 1 to 5. Participants in Study 3 were born before China's Reform and Opening policy of 1978. Bars = 95% confidence intervals. Each dot represents a participant.

The results were different for Chinese adults born after the Reform and Opening period. In this cohort, cultural priming did not affect internal attributions (Figure 7), *F*(2,255) = 1.10, *p =* .335, *f* ^*2*^ = .009 [.000, .033] (*M*_*China*_ = 2.89; *SD*_*China*_ = 0.42; *M*_*America*_ = 3.00; *SD*_*America*_ = 0.46; *M*_*Control*_ = 2.93; *SD*_*Control*_ = 0.45). The results were similar when controlling for all demographic variables. Culture priming did not influence internal attributions, *F*(2,250) = 0.56, *p* = .573, *f* ^2^ = .004 [.000, .022].

### Dialectical self‐concept

**FIGURE 8:**
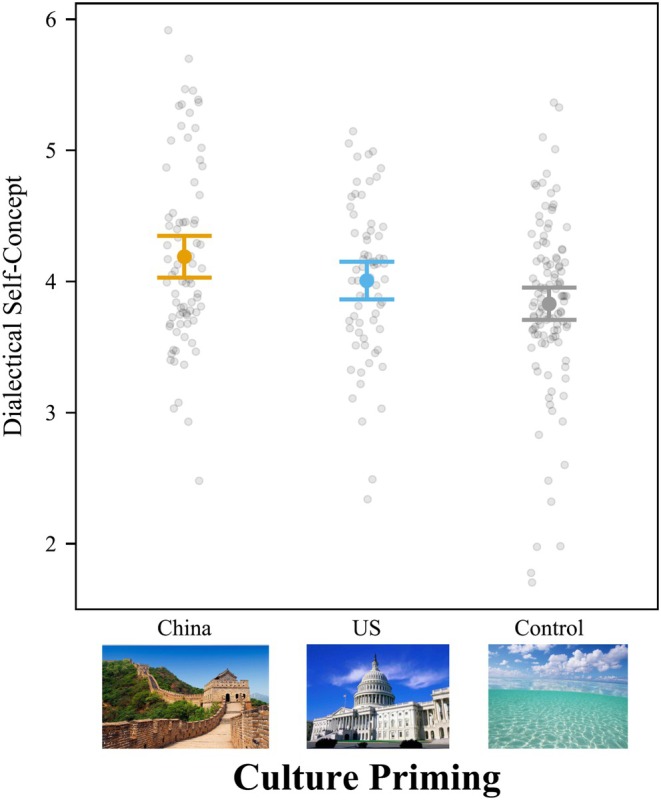
Priming US Culture Had No Effect on Dialectical Self‐Concept among Older Adults in Mainland China. Participants rated self‐concept questions from 1 to 7. Participants in Study 3 were born before China's Reform and Opening policy of 1978. Bars = 95% confidence intervals. Each dot represents a participant.

Cultural priming significantly affected dialectical self‐concept among older adults in China, *M*_*China*_ = 4.19; *SD*_*China*_ = 0.71; *M*_*America*_ = 4.01; *SD*_*America*_ = 0.59; *M*_*Control*_ = 3.83; *SD*_*Control*_ = 0.66, *F*(2,255) = 6.96, *p =* .001, *f* ^*2*^ = .055 [.014, .110]. The results were similar when controlling for all demographic variables, *F*(2,250) = 7.39, *p* < .001, *f* ^*2*^ = .059 [.016, .104]. But different from Hong Kong (Study 1) and Mainland college students (Study 2), older adults had significantly higher dialectical self‐concept with Chinese priming than neutral priming, *t*(255) = 3.72, *p* < .001, *d* = 0.548, 95% CI [0.201, 0.896]. The difference between the American and Chinese priming was not significant, *t*(255) = 1.68, *p* = .215, *d* = 0.279, 95% CI [−0.670, 0.112], and the difference between the American and neutral priming was also not significant, *t*(255) = 1.75, *p* = .188, *d* = 0.269, 95% CI [−0.093, 0.632] (Figure 8).

### Analysis combining the younger and older generations

Another way to test for generational differences is to put the younger generation sample from Study 2 and the older generation sample from Study 3 together in an analysis. Thus, we combined the samples and tested for an interaction effect between generation and cultural prime in a 3 (Culture Priming: China, America, control) × 2 (Generation: Younger vs. Older) ANOVA. This interaction tests whether the effect of the cultural primes was different for the older generation. The interaction effect was significant, *F*(2,524) = 5.09, *p* = .006, *f* ^*2*^ = .019 [.003, .043]. Simple effects analyses found that American cultural priming increased internal attributions for the younger generation, but not the older generation (Table 4).

**TABLE 4:** Simple‐effect analyses for internal attributions.

Contrast	Difference	*SE*	*DF*	*t*	*p*	Cohen's *d* [95% CI]
Younger Generation						
America‐China	0.37	0.06	524	6.25	<.001	0.939 [0.585, 1.292]
America‐Control	0.13	0.06	524	2.19	.074	0.330 [−0.024, 0.684]
Control‐China	0.24	0.06	524	4.18	<.001	0.608 [0.267, 0.950]
Older Generation						
America‐China	0.11	0.06	524	1.63	.235	0.270 [−0.120, 0.660]
America‐Control	0.07	0.06	524	1.13	.497	0.174 [−0.188, 0.535]
Control‐China	0.04	0.06	524	0.66	.790	0.097 [−0.250, 0.443]

*Note*: This analysis combines the younger generation sample from Study 2 and the older generation sample from Study 3 to test whether the effect of cultural priming differed between the two samples.

Results were similar for the combined analysis on dialectical self‐concept. The interaction effect was significant, *F*(2,524) = 6.90, *p* = .001, *f* ^*2*^ = .027 [.007, .053]. Simple effects analyses found that the college students primed with American culture had higher dialectical self‐concept ratings than those primed with Chinese culture, although the difference was not significant. In contrast, the older generation primed with Chinese culture had similar dialectical self‐concept ratings to those primed with American (Table 5).

**TABLE 5:** Simple‐effect analyses for dialectical self‐concept.

Contrast	Difference	*SE*	*DF*	*t*	*p*	Cohen's *d* [95% CI]
Younger Generation						
America‐China	0.23	0.09	524	2.55	.030	0.383 [0.030, 0.736]
America‐Control	0.17	0.09	524	1.89	.142	0.285 [−0.069, 0.639]
Control‐China	0.06	0.09	524	0.67	.779	0.098 [−0.244, 0.440]
Older Generation						
America‐China	−0.18	0.10	524	−1.83	.161	0.304 [−0.693, 0.086]
America‐Control	0.18	0.09	524	1.91	.137	0.293 [−0.068, 0.655]
Control‐China	−0.36	0.09	524	−4.05	<.001	0.597 [0.250, 0.944]

*Note*: This analysis combines the younger generation sample from Study 2 and the older generation sample from Study 3 to test whether the effect of cultural priming differed between the two samples.

## DISCUSSION

Study 3 found no significant effects of cultural priming on older Chinese Mainland adults, who were born before China's Reform and Opening. This suggests that older Chinese adults are not bicultural, which fits with the explanation that they grew up in an era when China was less open to the outside world. An analysis combining the younger generation and older generation samples found a significant interaction effect, suggesting that the effect of cultural priming was different between the two generations.

## GENERAL DISCUSSION

In general, our results replicated the findings of earlier East–West differences (Morris & Peng, 1994) and studies of bicultural identity (Hong et al., 1997, 2000) over 20 years later. Study 1 found evidence that students in Hong Kong are bicultural. They agreed more with internal attributions after priming with American cultural icons than Chinese cultural icons. They also saw themselves as more dialectical after priming with American culture.

In Study 2, we tested whether college students in Mainland China are also bicultural, like participants in Hong Kong. The results suggested that students in Mainland China are bicultural. American cultural priming increased internal attributions and dialectical self‐concept. Chinese cultural priming increased external attributions (Data S1).

In Study 3, we also tested for generational differences by comparing young college students and older adults born before China's Reform and Opening. The older generation did not show bicultural patterns in attributions like the other groups. This suggests that biculturalism is a recent phenomenon in China, as Chinese society has become more and more open to the outside world.

These results also demonstrate an important boundary condition for biculturalism theory. By sampling a population that has less exposure to multiple cultures, we could test the idea that cultural exposure matters. We need “negative controls” like this age cohort in China to truly know whether bicultural exposure truly differentiates people who respond to bicultural primes and people who do not.

## NEW EVIDENCE FOR BICULTURALISM IN MAINLAND CHINA

Contemporary Chinese college students have been living in China's era of globalisation since they were born. This means they have experienced foreign cultures in a way that older generations did not. This can explain why priming with cultural symbols was able to elicit behaviours similar to people from different cultures (Chiu & Hong, 2018).

This study adds to prior findings in several ways. First, previous research has found evidence that people in Hong Kong are bicultural (Hong et al., 2000). This makes sense with the fact that Hong Kong is a Chinese area with a history of British colonization. Hong Kong has also been an international hub for years, long before Mainland China opened up. Yet our study found evidence that college students in Mainland China also show evidence of bicultural identities.

Second, we tested two generations from China: college students and older adults born before China's Reform and Opening in 1978. Cultural priming had no effect on older adults' attributions. This is consistent with the idea that China was more closed at that time and people had less exposure to outside cultures. This helps sketch out boundary conditions for bicultural identity.

## CULTURAL INFLUENCES ON CAUSAL EXPLANATION

This study found evidence that cultural priming influences people's causal explanations. Priming Chinese culture increased people's external attributions—explanations to the situation, environment, or other people. In contrast, priming American culture increased people's internal attributions—explanations to the actor's internal motivations or personality.

Studies have found that people in China attribute more to social circumstances and relationships than Americans (Bond, 1983; Morris & Peng, 1994). When describing people, Westerners tended to give decontextualised descriptions of dispositions that were stable across situations than people from India and Africa (Shweder & Bourne, 1982; Valchev et al., 2013). This study adds to the growing evidence that people's attribution style can also change across situations.

## A SURPRISING FINDING: PRIMING AMERICAN CULTURE INCREASES DIALECTICISM?

Because people in China tend to score higher on dialectical thinking (Peng & Nisbett, 1999), it would be logical to predict that priming Chinese culture would increase people's dialectical self‐concept. In contrast, priming US culture should lower dialectical self‐concept. Yet Study 2 found the opposite. Priming American culture increased dialectical self‐concept.

One possible explanation is that priming American culture reminded the Chinese students of the contradictory influences in their lives. Previous studies have found that priming Chinese students to think about their experience studying abroad increased dialectical thinking (increased tolerance of contradiction; Hu et al., 2021). This multicultural experience may give people more experience with differences, change, and contradiction. Although dialectical thought is a traditional Chinese pattern of thought, it may also be enhanced by broadening people's perspectives and experiences.

Biculturalism is also consistent with theories of how people can hold multiple conflicting identities (e.g., Singelis, 1994; Triandis, 1989). For example, the Taiji Model of Self is based on the idea of yin‐yang (Kitayama & Markus, 1999; Wang & Wang, 2021). Yin‐yang is the idea that (1) things are constantly changing and (2) things have contradictory components. In their classic paper on independent and interdependent selves, Markus and Kitayama (2010) argued that people have both interdependent and independent self‐concepts. But cultures typically promote the development of one or the other self more strongly.

Despite the low reliability of the Dialectical Self‐Concept Scale in our studies, we chose to include it for two reasons. (1) Although it was not the major focus of the present research, it presents an intriguing possibility: biculturalism may increase dialectical thinking because exposure to different cultures may require heightened sensitivity to things that differ from people's primary culture. This suggests an intriguing direction for future research.

(2) Previous research has argued that dialectical thought can help explain cultural differences (e.g., Nisbett, 2003; Spencer‐Rodgers et al., 2009, 2012). People in East Asia are more likely to believe that people can change and behave in contradictory ways across situations. Some researchers have argued that differences in attribution style alone cannot explain cultural differences in cognition (Spencer‐Rodgers et al., 2012). They argue that dialectical thought more generally can explain more broadly than attribution style alone. However, this reasoning has not been directly tested. None of our studies found a link between internal attribution and dialecticism (at least as measured by this self‐report scale). This is an important issue that warrants further exploration.

(3) Finally, including the scale results allows for comparisons with prior research. It may also guide future research. For example, reporting the low reliability is important to have on the record. It may inspire future researchers to edit the scale to improve the reliability. Thus, we think there is value in presenting the results of the Dialectical Self‐Concept Scale, even though the reliability was below conventions.

## LIMITATIONS AND FUTURE DIRECTIONS

Our study has several limitations worth noting. First, we found evidence that priming American culture increased dialectical self‐concept, but this finding was not consistent across all groups. However, it does fit with a previous study, which found that priming Chinese students to think about their study abroad experiences increased dialectical thinking (Hu et al., 2021). This is surprising because many Chinese students study abroad in Western cultures, and Western cultures are known for thinking less dialectically (Peng & Nisbett, 1999). This finding emphasises an interesting question for future research: Does biculturalism generally increase dialectical thinking, even if the second culture is less dialectical than the first culture?

One explanation is that exposure to different cultures confronts people with contradictions between their first and second cultures. However, future research could test rival explanations. For instance, do American biculturals living abroad outside of Asia think more dialectically?

A second limitation is the psychometric properties of the scales. We used the attribution style measure in line with prior research, but it has not been tested extensively as a psychometric scale. Also, we corrected the reliability of the Dialectical Self‐Concept Scale for acquiescent response style, as the reliability of the original scale was unsatisfactory. Future research should focus on developing scales or paradigms that better align with psychometric standards to measure the variables of interest.

A third limitation is that most of our participants were students. This limits the generalisability of our findings. Future studies can test these ideas with more diverse samples in China and in other countries with multiple cultural influences, such as India and Mexico.

Finally, a limitation is that our comparison of college students and older adults cannot rule out age effects. We interpret the lack of priming effects in the older adults as consistent with the explanation that they grew up in a more closed society. However, it is possible that this is an age effect. Perhaps older people are less sensitive to priming or have less practice in activating their bicultural identities.

## CONCLUSIONS

The results of this study support the theoretical framework of cultural frame‐switching. The results validate previous studies finding that people in Hong Kong are bicultural and extend these findings to students in Mainland China.

For the distinction of innate biculturalism versus achieved biculturalism, we suspect that the concept of innate biculturalism applies better to Hong Kong residents because they experience early immersive culture mixing (Martin & Shao, 2016). However, in Mainland China, we suspect that most biculturals receive their exposure to other cultures outside the home environment or later in life, which would count as achieved biculturalism (Martin & Shao, 2016). Achieved biculturalism is a fast‐growing demographic around the world. This makes it more important to examine how multicultural experiences shape cognitive processes. Our study offers a small step in this direction by testing participants across two societies and across generations.

The findings serve as a reminder that cultural differences in thought style are not fixed. Instead, cultural patterns can adapt to the context. Temporarily priming cultural identities can shift people's causal explanations and dialectical thinking.

But not all biculturals adhere to cultural norms. Some researchers have argued that some bicultural people feel secure with their two identities, whereas others feel more conflict between their identities (Amiot et al., 2007). One study found that people who feel conflict between their identities sometimes respond in ways that contradict cultural norms, which may be related to less openness and more neuroticism (Mok, 2022).

These results help us understand people's complex cultural identities. People can activate different cultural thought styles. However, our test with a negative control group (the older generation) offers a crucial test of the idea that cultural exposure is necessary for people to respond to cultural priming.

## AUTHOR CONTRIBUTIONS

**Yi‐meng Wang:** Data curation; investigation; writing – original draft; writing – review and editing; methodology; formal analysis. **Feng‐yan Wang:** Conceptualization; writing – original draft; supervision; investigation. **Thomas Talhelm:** Supervision; validation; visualization; writing – original draft; writing – review and editing. **Yi‐qun Chen:** Data curation; investigation.

## FUNDING INFORMATION

Feng‐yan Wang received funding from Grant No. 31971014 from the National Natural Science Foundation of China. Yi‐meng Wang received funding from the China Scholarship Council (CSC) during a visit to the University of Chicago.

## CONFLICT OF INTEREST STATEMENT

The authors declare that they have no conflicts of interest.

## ETHICS STATEMENT

The studies involving human participants were reviewed and approved by the Research Ethics Committee at Nanjing Normal University. The participants gave informed consent online.

## Supporting information


Data S1.

## Data Availability

The study design, hypotheses, and analysis plans were not preregistered. Measures and coding schemes used in this study were compiled from or informed by peer‐reviewed literature. A full list of survey items and additional information regarding our data and coding are available at https://osf.io/u26zh/?view_only=b8c5801c7c2d4ebf8c3d9fb85884258a.
