# Supplementary material for: New evidence finds young people in Mainland China are now bicultural
Source: Br J Psychol. 2025 Jan 13;116(2):460–83. doi: 10.1111/bjop.12767 (PMC11984341; doi:10.1111/bjop.12767)
Supplement: Supplementary file 1 — Data S1. [file BJOP-116-460-s001.docx]

# Supplemental Materials

**Table S1**

*Descriptive Statistics of the Samples in This Study and the Original Study*

|  | Study 1 (Hong Kong) | | | Study 2 (Mainland) | | Study 3 (Mainland) | |
| --- | --- | --- | --- | --- | --- | --- | --- |
| Variable | Hong et al. (1997) | Replication | | College Students | | Older Adults | |
|  |  | Full | Final | Full | Final | Full | Final |
| **Sample Size** | 75 | 120 | 107 | 300 | 272 | 300 | 258 |
| Chinese Culture | 25 | 38 | 36 | 102 | 95 | 98 | 78 |
| American Culture | 25 | 40 | 35 | 105 | 83 | 75 | 68 |
| Neutral/Landscape | 25 | 42 | 36 | 93 | 94 | 127 | 112 |
| **Age** |  |  |  |  |  |  |  |
| *M*±*SD* |  | 21.43±3.32 | 21.41±3.26 | 22.72±4.07 | 22.37±3.58 | 52.81±9.24 | 55.70±5.18 |
| Range |  | 18-34 | 18-34 | 17-38 | 17-38 | 22-70 | 43-70 |
| **Gender** |  |  |  |  |  |  |  |
| Female |  | 63.3% | 64.5% | 64.7% | 62.9% | 44.3% | 45.7% |
| Male |  | 36.7% | 35.5% | 35.3% | 37.1% | 55.7% | 54.3% |
| **Education**  **(Bachelor or higher %)** | | 93.4% | 93.3% | 90.0% | 97.1% | 58.3% | 60.5% |
| **Study Abroad %** |  | 5.6% | 5.6% | 7.7% | 8.5% | 4.7% | 5.4% |

*Note:* *The final sample is after exclusions, such as the attention check question. The study abroad question asks students whether they have studied abroad.

**Section 1: Results Separating Internal and External Attribution Questions**

In the main text, we report on the analyses conducted by combining the internal attribution indicators calculated for all items. Here we report analyses by separating out three types of items. We tested whether separating these items would make a difference because it is not clear whether (1) internal and external attributions are opposite ends of a single continuum or (2) internal and external attributions are separate, such that some people endorse both at the same time. Thus, in this section, we test whether the results are similar if we separate these items with full sample.

**External questions:**These questions just ask about external forces (Items 2, 4, and 5);

**Internal question:** This question just asks about internal forces (Item 3);

**Continuum question:** This question asks about a continuum from internal to external (Item 1).

1. “Why do you think there is one fishing swimming in front of/behind the group of fish?” Participants responded from 1 (*very confident that it is because the one fish is leading/independent from the other fish* [an internal cause]) to 5 (*very confident that it is because the one fish is being chased/rejected by the other fish* [an external cause]).

2. “Is the fish on the left side an important member of the school on the right?” Participants responded from 1 (*I think the fish on the left is absolutely not an important member of the school on the left* [an internal attribution]) to 5 (*I think the fish on the left is absolutely an important member of the school on the left* [an external attribution]).

3. “How much is the fish on the left influenced by its own factors?” Participants responded from 1 (*I think the fish on the left’s influence* *from its own internal factors is extremely small* [an external attribution]) to 5 (*I think the fish on the left’s influence* *from its own internal factors is extremely large* [an internal attribution]).

4. “How much is the fish on the left influenced by the other fish?” Participants responded from 1 (*I think the fish on the left’s influence* *from the other fish is extremely small* [an internal attribution]) to 5 (*I think the fish on the left’s influence* *from the other fish is extremely large* [an external attribution]).

5. “To what extent are the actions of the other fish influenced by the fish on the right?” Participants responded from 1 (*I think the other fish’s influence* *from the fish on the right is extremely small* [an internal attribution]) to 5 (*I think the other fish’s influence* *from the fish on the right is extremely large* [an external attribution]).

The results were largely similar regardless of whether we combined all these items or separated them. This suggests that people tend to think of internal and external attributions as opposite ends of a continuum.

## Study 1

We ran one-way ANVOAs across the three conditions (1 = Chinese prime, 2 = American prime, 3 = neutral prime). Table S2 presents the results.

**Internal Question:** Cultural priming significantly affected internal attributions, *F*(2, 117) = 17.34, *p <* .001, *f^2^* = .297 [.138, .488] without covariates, *F*(2, 112) = 16.16, *p <* .001, *f^2^* = .289 [.129, .484] and with covariates (Figure S1).

**External Questions:** Cultural priming significantly affect external attributions without covariates, *F*(2, 117) = 3.85, *p =* .024, *f^2^* = .066 [.005, .157], and with covariates, *F*(2, 112) = 4.90, *p =* .009, *f^2^* = .086 [.012, .195](Figure S2).

**Continuum Question:** Cultural priming significantly affected the continuum question without covariates, *F*(2, 117) = 4.21, *p = .*017, *f^2^* = .071 [.007, .168], and marginally significantly with covariates, *F*(2, 112) = 2.76, *p = .*068, *f^2^* = .049 [.000, .131](Figure S3).

**Table S2**

*The One-Way ANOVA on Attributions (Separate) and Dialectical Self-Concept for Study 1*

|  | Culture Priming | | | *F* | *p* | *f*^2^ |
| --- | --- | --- | --- | --- | --- | --- |
|  | China | America | Control |  |  |  |
| Internal Attributions (Item 3) | 2.51±0.81^a^ | 3.42±0.66^b^ | 3.15±0.73^b^ | 17.34 | < .001 | .297 |
| External Attributions (Items 2, 4, 5) | 3.27±0.66^b^ | 2.94±0.53^a^ | 3.03±0.44^ab^ | 3.85 | .024 | .066 |
| Continuum Question (Item 1) | 3.00±0.63^b^ | 2.63±0.60^a^ | 2.70±0.58^a^ | 4.21 | .017 | .071 |

*Note*: Distinct letters indicate statistically significant differences. Letters that are the same denote no significant difference.

**Figure S1**

*Participants Primed with American Culture Agreed More with Internal Attributions*


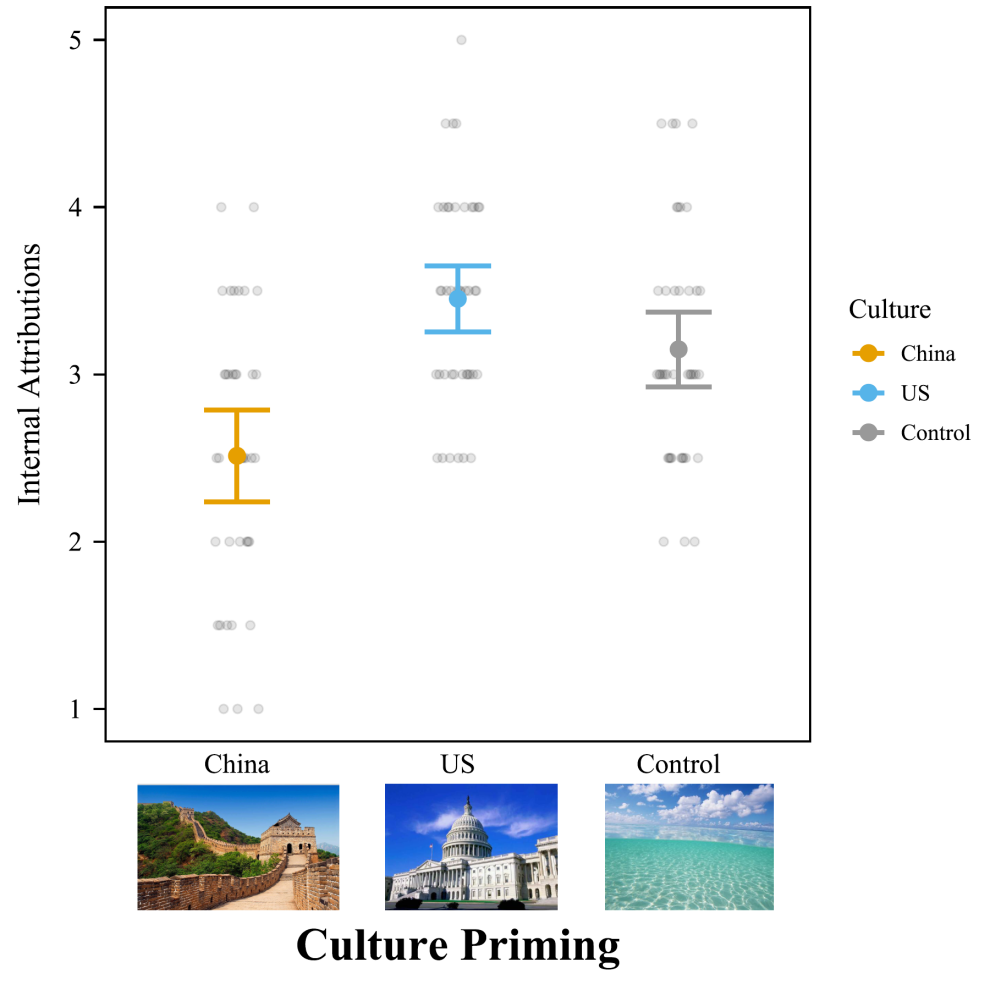


*Note.* Participants rated internal attributions from 1 to 5 (*How much is the fish on the left influenced by its own factors*). The difference between US cultural priming and the control condition was not significant. Bars = 95% confidence intervals. Each dot represents a participant.

**Figure S2**

*Participants Primed with Chinese Culture Agreed More with External Attributions*


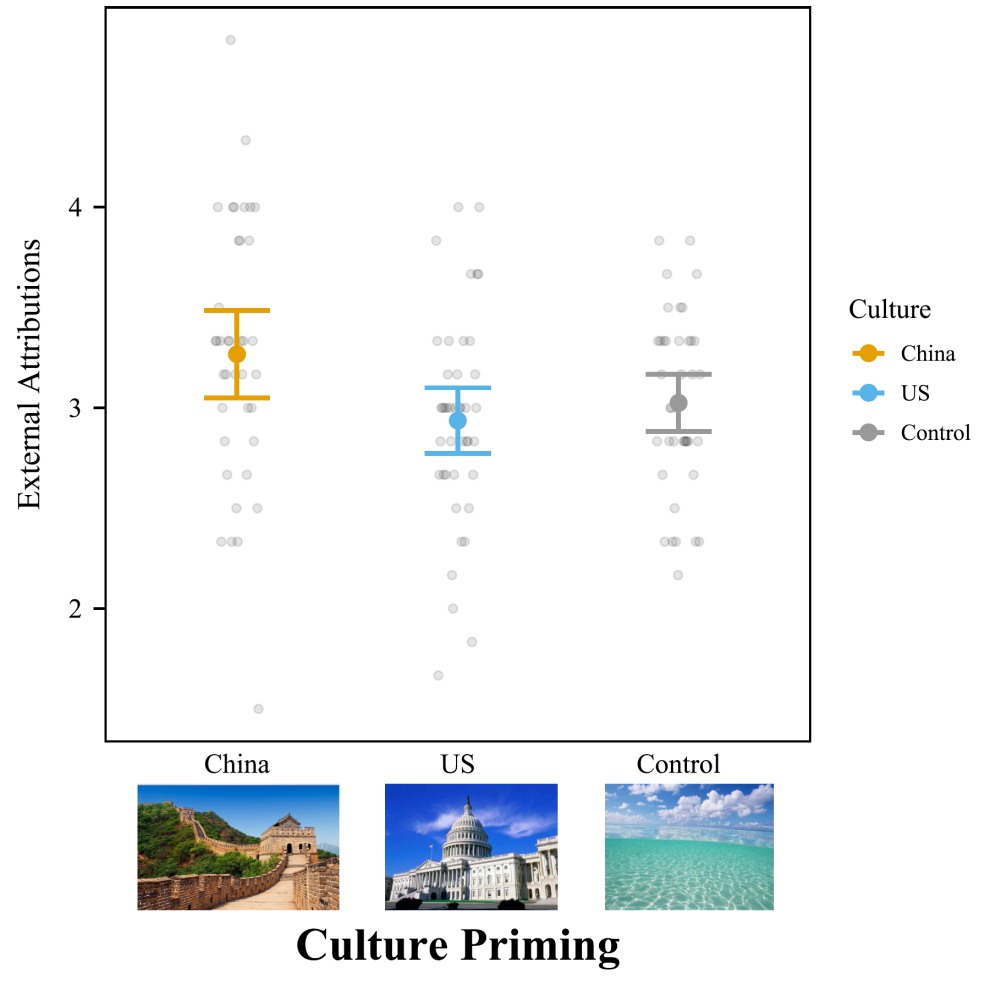


*Note.* Participants rated external attributions from 1 to 5 on items 2, 4, 5. The difference between US cultural priming and the control condition was not significant, nor was the difference between Chinese cultural priming and the control condition. Bars = 95% confidence intervals. Each dot represents a participant.

**Figure S3**

*Participants Primed with Chinese Culture Agreed More with External Attributions*

*
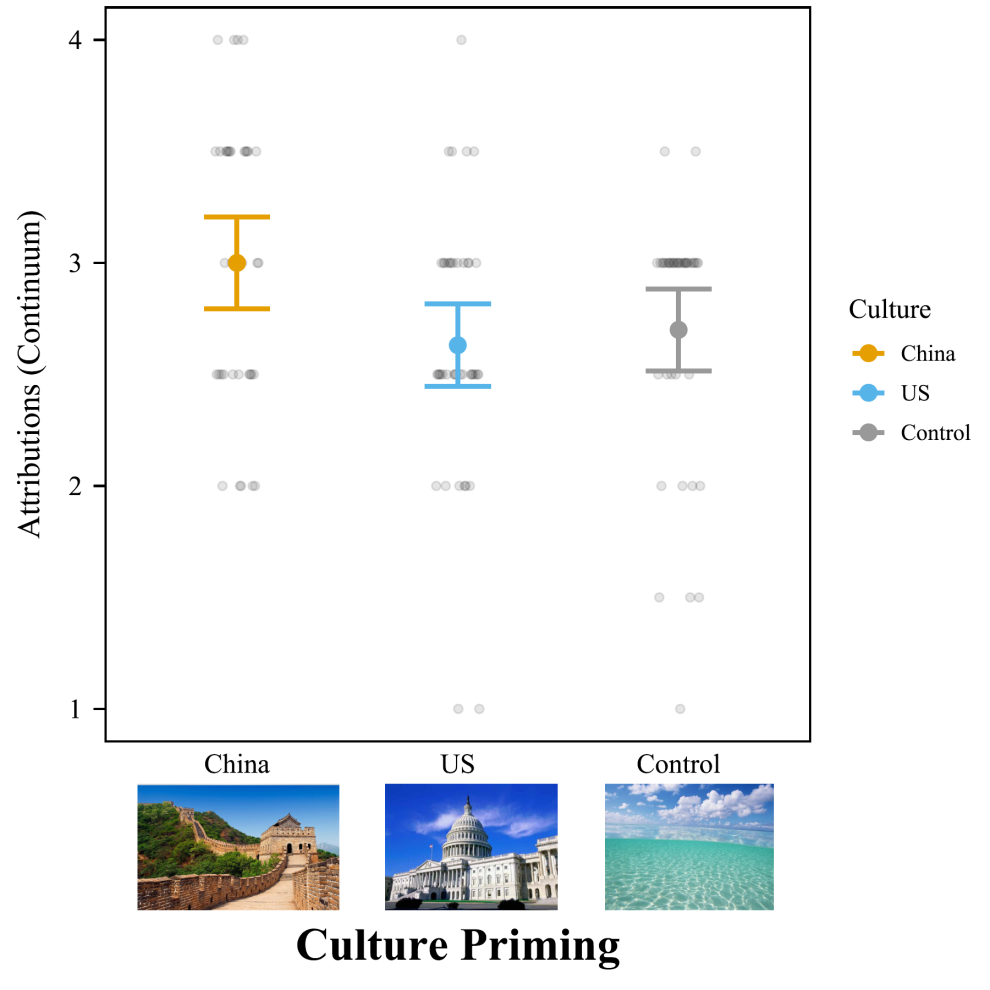
*

*Note.* Participants rated attributions on a continuum from 1 (*internal*) to 5 (*external*) on Item 1. The difference between American cultural priming and the control condition was not significant. The difference between Chinese cultural priming and the control condition was marginally significant. Bars = 95% confidence intervals. Each dot represents a participant.

## Study 2

**Internal Question:** Cultural priming significantly affected internal attributions, without covariates *F*(2, 297) = 9.66, *p <* .001, *f^2^* = .065 [.022, .120], and with covariates *F*(2, 292) = 3.27, *p =* .039, *f^2^* = .022 [.001, .056](Figure S4).

**External Questions:** Cultural priming significantly affected external attributions, without covariates, *F*(2, 297) = 10.07, *p < .*001, *f^2^* = .068 [.024, .124], and with covariates, *F*(2, 292) = 9.24, *p < .*001, *f^2^* = .064 [.020, .117](Figure S5).

**Continuum Question:** Cultural priming significantly affected responses to the continuum question without covariates, *F*(2, 297) = 16.73, *p < .*001, *f^2^* = .112 [.054, .183], and with covariates, *F*(2, 292) = 5.52, *p = .*004, *f^2^* = .037 [.007, .081](Figure S6).

**Table S3**

*One-Way ANOVA on Attributions (Separate) for Study 2*

|  | Culture Priming | | | *F* | *p* | *f*^2^ |
| --- | --- | --- | --- | --- | --- | --- |
|  | China | America | Control |  |  |  |
| Internal Attributions (Item 3) | 2.52±0.84^a^ | 2.93±0.82^b^ | 2.45±0.80^a^ | 9.66 | < .001 | .065 |
| External Attributions (Item 2,4,5) | 3.00±0.50^b^ | 2.76±0.53^a^ | 2.71±0.45^a^ | 10.07 | < .001 | .068 |
| Continuum Question (Item 1) | 2.55±0.75^b^ | 2.01±0.64^a^ | 2.15±0.67^a^ | 16.73 | < .001 | .112 |

*Note*: Distinct letters indicate statistically significant differences. Letters that are the same denote no significant difference.

**Figure S4**

*Participants Primed with American Culture Agreed More with Internal Attributions*


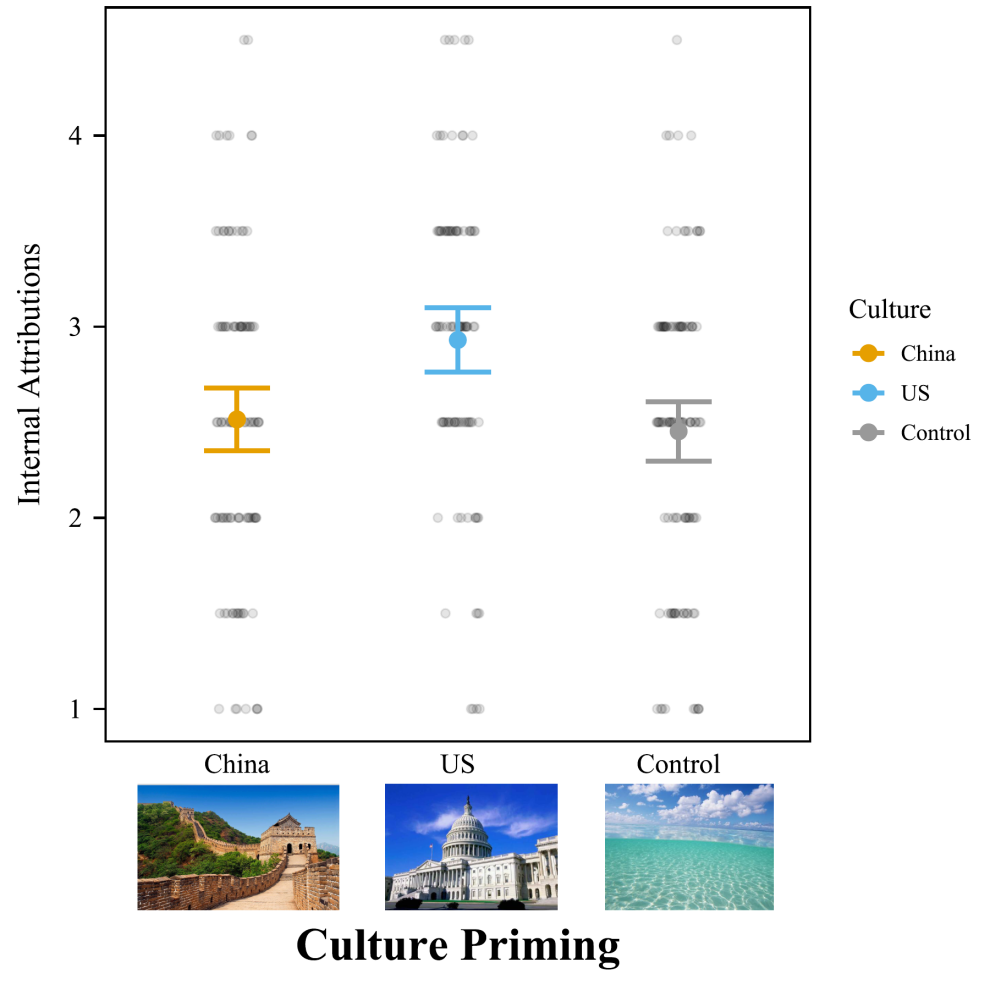


*Note.* Participants rated internal attributions from 1 to 5 on item 3 (*How much is the fish on the left influenced by its own factors*) The difference between Chinese cultural priming and the control condition was not significant. Bars = 95% confidence intervals. Each dot represents a participant.

**Figure S5**

*Participants Primed with Chinese Culture Agreed More with External Attributions*


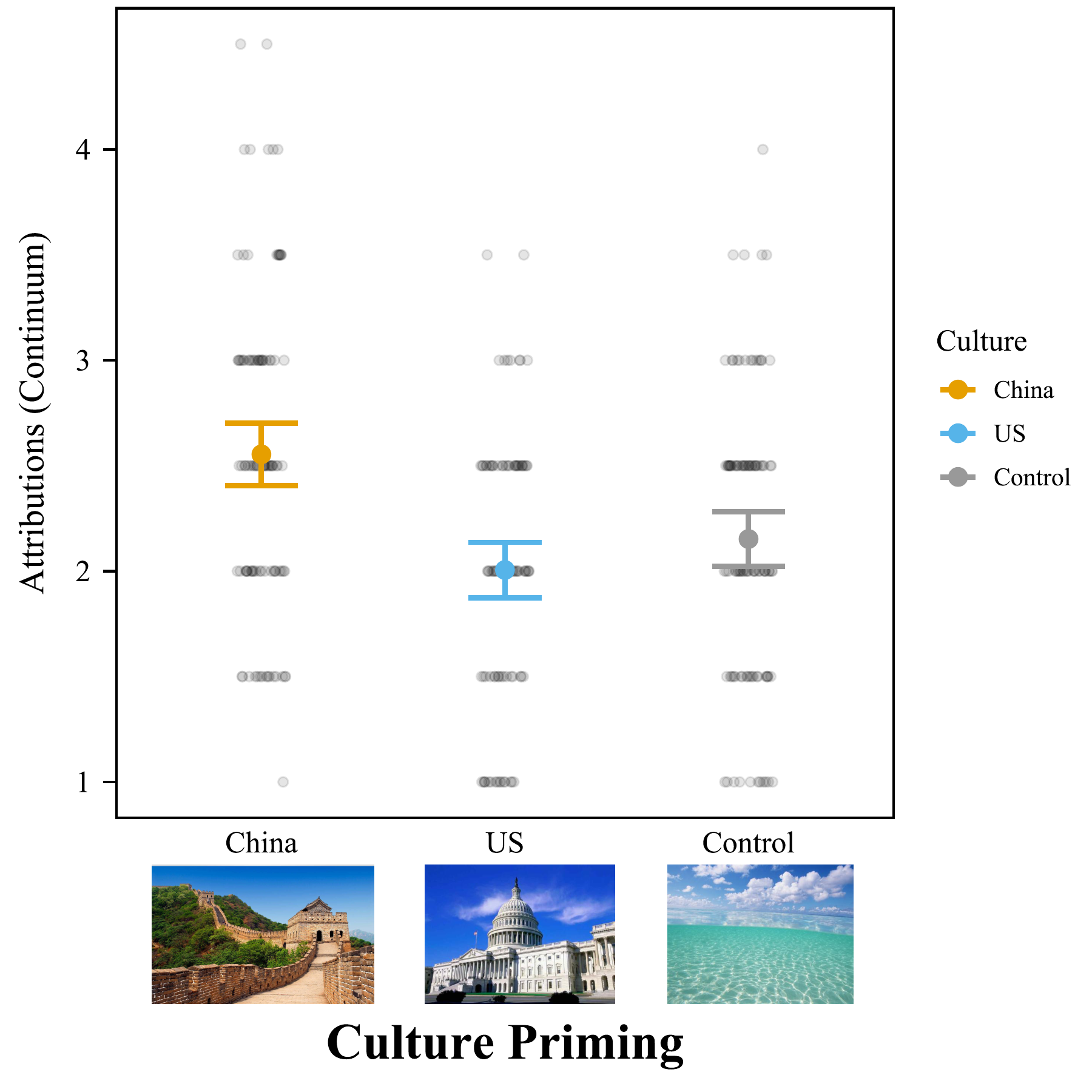


*Note.* Participants rated external attributions from 1 to 5 on items 2, 4, and 5. The difference between American cultural priming and the control condition was not significant. Bars = 95% confidence intervals. Each dot represents a participant.

**Figure S6**

*Participants Primed with Chinese Culture Agreed More with External Attributions*

*
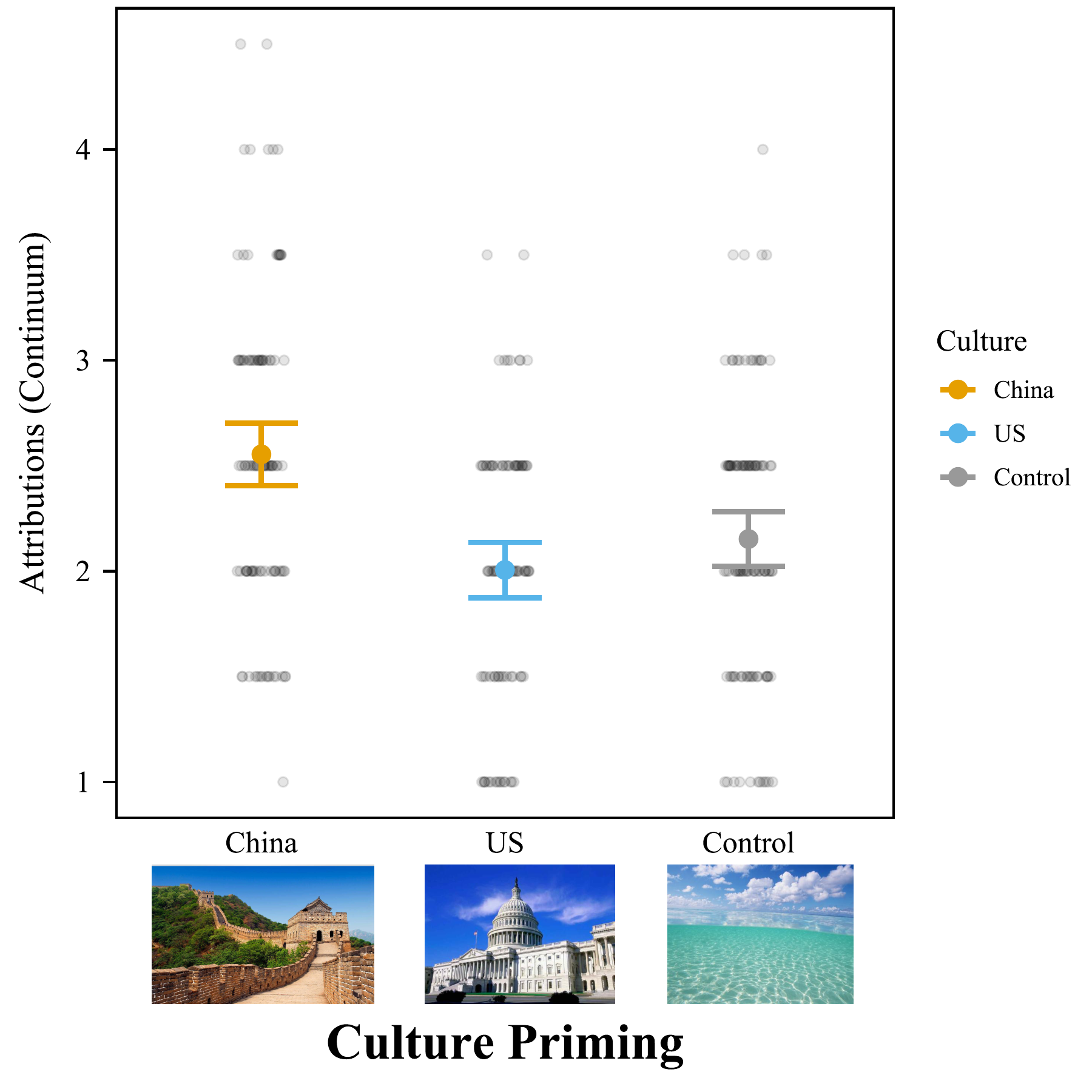
*

*Note.* Participants rated attributions in a continuum from 1 (*internal*) to 5 (*external*) on Item 1. The difference between American cultural priming and the control condition was not significant. Bars = 95% confidence intervals. Each dot represents a participant.

## Study 3

**Internal Question:** Cultural priming did not significantly affect internal attributions among older adults in China without covariates, *F*(2, 297) = 9.46, *p =* .632, *f^2^* = .003 [.000, .017], or with covariates, *F*(2, 292) = 0.86, *p =* .425, *f^2^* = .006 [.000, .025] (Figure S7).

**External Questions:** Cultural priming did not significantly affect external attributions without covariates, *F*(2, 297) = 1.58, *p =* .208, *f^2^* = .011 [.000, .035], or with covariates, *F*(2, 292) = 0.21, *p =* .812, *f^2^* <.001 [.000, .010] (Figure S8).

**Continuum Question:** Cultural priming did not affect responses to the continuum question without covariates, *F*(2, 297) = 0.09, *p =* .915, *f^2^* = .001 [.000, .004], or with covariates, *F*(2, 292) = 0.21, *p =* .812, *f^2^* = .001[.000, .010] (Figure S9).

**Table S4**

*ANOVA on Attributions (Separate) for Study 3*

|  | Culture Priming | | | | | | *F* | *p* | *f*^2^ |
| --- | --- | --- | --- | --- | --- | --- | --- | --- | --- |
|  | China | | America | | Control | |  |  |  |
| Internal Attributions (Item 3) | | 3.04±0.97^a^ | | 3.15±0.93^a^ | | 3.15±0.86^a^ | 0.46 | .632 | .003 |
| External Attributions (Item 2,4,5) | | 3.20±0.63^a^ | | 3.05±0.61^a^ | | 3.08±0.62^a^ | 1.58 | .208 | .011 |
| Continuum Question (Item 1) | | 2.50±0.74^a^ | | 2.55±0.96^a^ | | 2.52±0.84^a^ | 0.09 | .915 | .001 |

*Note*: Distinct letters indicate statistically significant differences. Letters that are the same denote no significant difference.

**Figure S7**

*Culture Priming Did Not Affect Internal Attributions for Older Adults Born Before Reform and Opening*


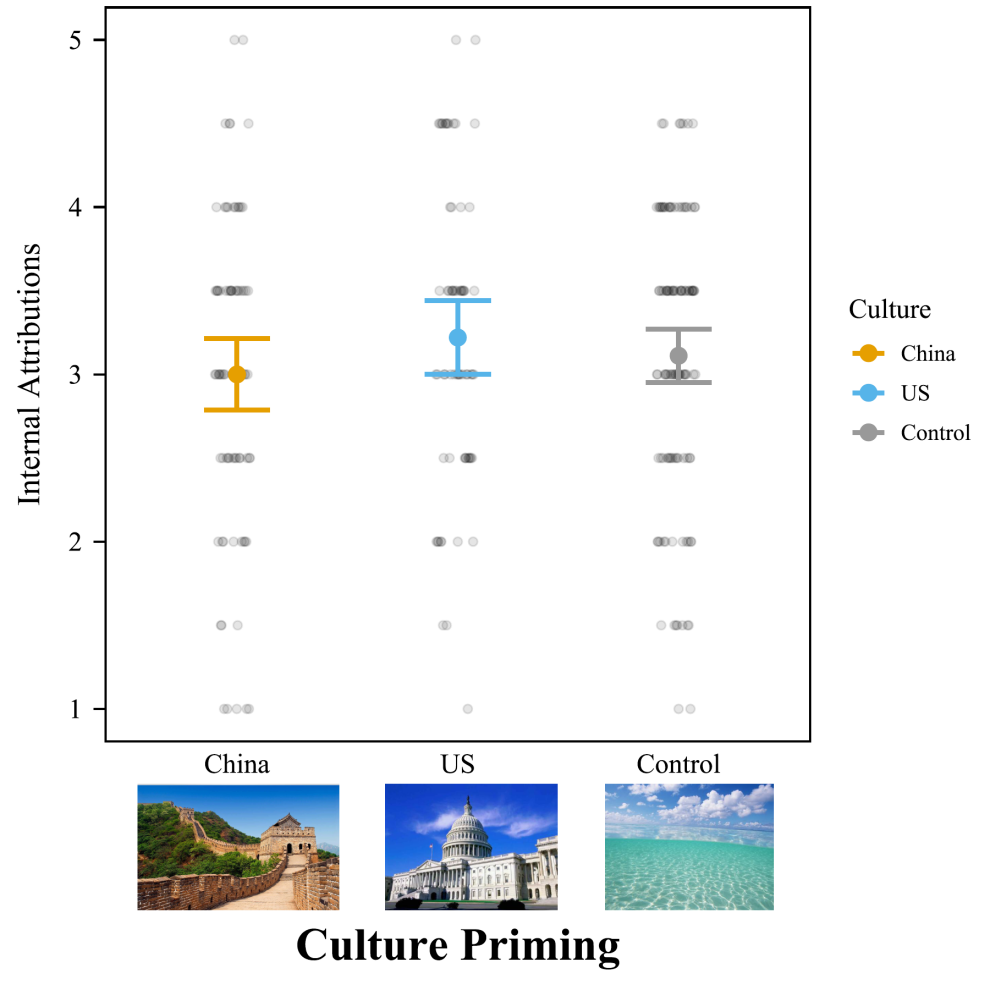


*Note.* Participants rated internal attributions from 1 to 5 on Item 3 (*How much is the fish on the left influenced by its own factors*). Bars = 95% confidence intervals. Each dot represents a participant.

**Figure S8**

*Culture Priming Did Not Affect External Attributions for Older Adults Born Before Reform and Opening*


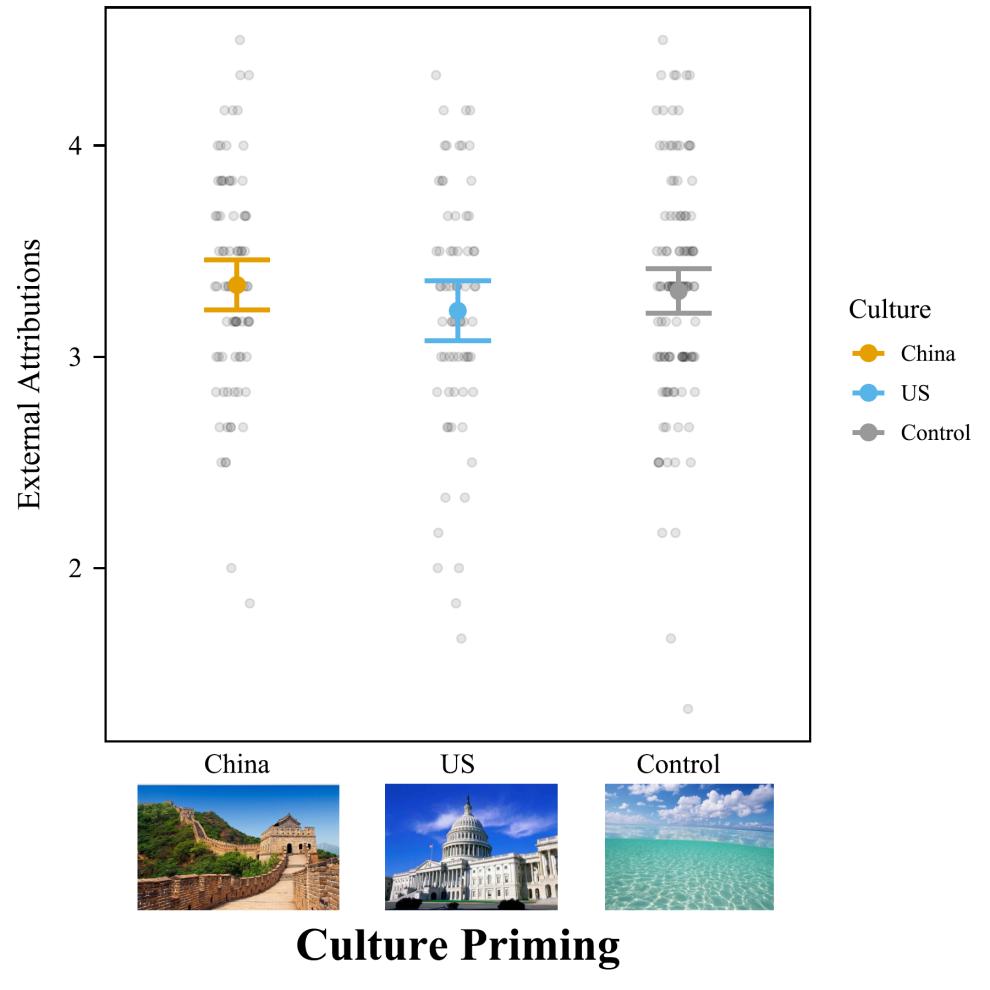


*Note.* Participants rated internal attributions from 1 to 5 on items 2, 4, and 5. Bars = 95% confidence intervals. Each dot represents a participant.

**Figure S9**

*Culture Priming Did Not Affect Attributions for Older Adults Born Before Reform and Opening*


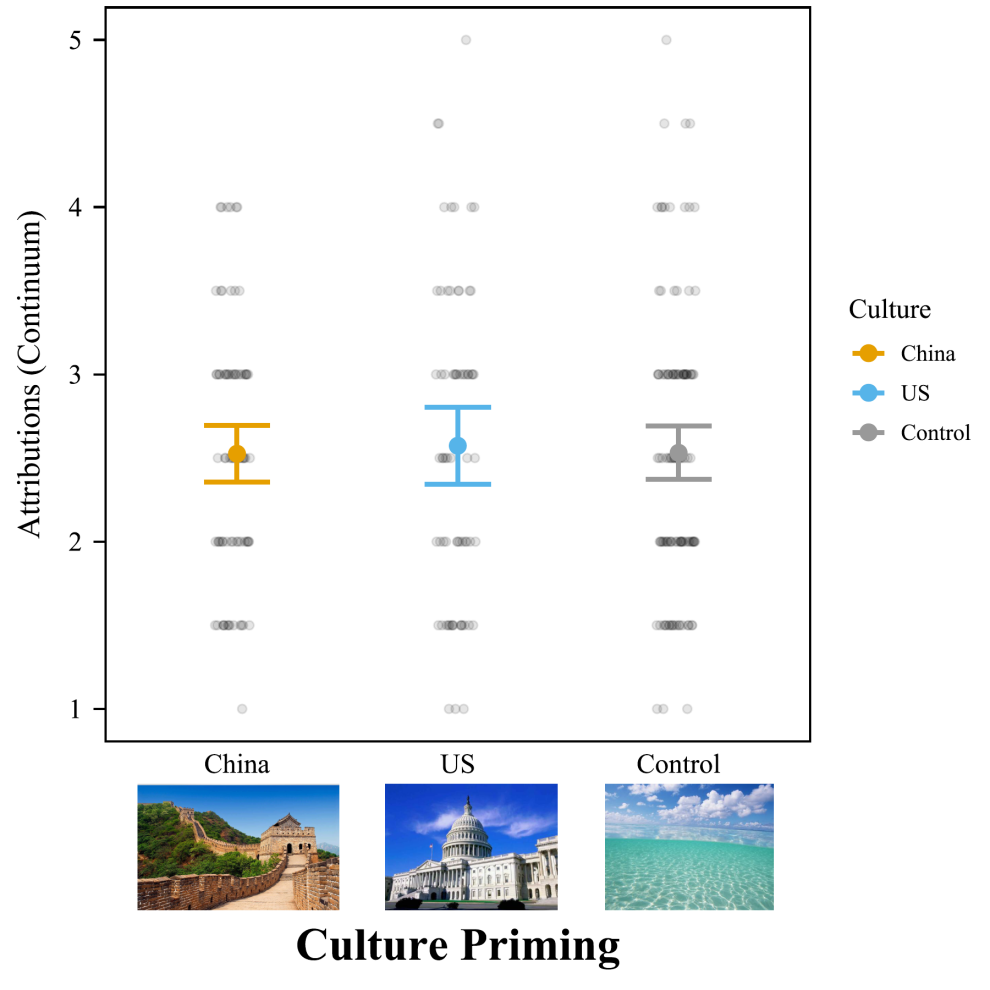


*Note.* Participants rated attributions on a continuum from 1 (*internal*) to 5 (*external*) on Item 1. Bars = 95% confidence intervals. Each dot represents a participant.

**Section 2: Results for the Original Dialectical Self-Scale**

In this section, we test whether the results are similar using the final sample with dialectical self scale scores that have not undergone acquiescent response correction and without excluding low-loading items.

## Study 1:

Culture priming had a marginally significant effect on dialectical self-concept, *F*(2,104) = 2.98, *p =* .055, *f^2^* = .06 without control variables (Figure S10). Controlling for all demographics, the main effect of culture priming on dialectical self-concept was not significant, *F*(2,99) = 1.86, *p =* .160, *f*^2^ = .04. Surprisingly, post-hoc tests showed priming with American culture increased dialectical self-concept ratings (*M* = 4.56; *SD* = 0.60) over the neutral priming (*M* = 4.29; *SD* = 0.38, *t*[104] = 2.44, *p =* .043, *d* = 0.58). The difference between the American and Chinese priming was not significant (*M* = 4.43; *SD* = 0.45, *t*[104] = 1.16, *p =* .480, *d* = 0.28).

**Figure S10**

*American Culture Priming Increased Dialectical Self-Concept Over the Neutral Priming*

**
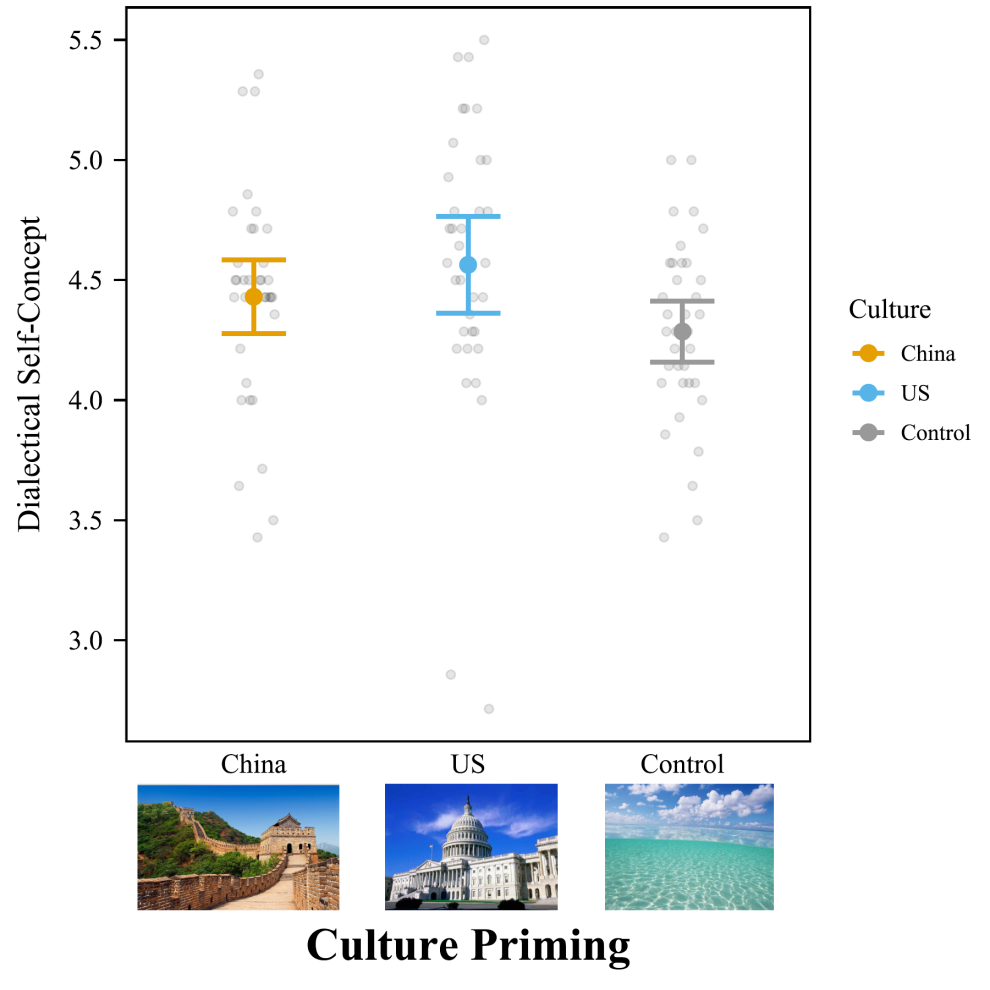
**

*Note:* Participants rated 14 items on self-concept from 1 (*strongly disagree*) to 7 (*strongly agree*), such as “I sometimes believe two things that contradict each other.” Bars = 95% confidence intervals. Each dot represents a participant.

## Study 2:

Cultural priming significantly influenced college students’ dialectical self-concept, *F*(2,269) = 4.34, *p =* .014, *f^2^* = .03 (Figure S11). Like Study 1, priming college students with American pictures led to higher ratings of dialectical self-concept (*M* = 4.82; *SD* = 0.52) than priming with Chinese pictures (*M* = 4.60; *SD* = 0.47), *t*(269) = 2.93, *p* = .010, *d* = 0.44. American cultural priming also led to similar dialectical self-concept to neutral priming, *M* = 4.68; *SD* = 0.49, *t*(269) = 1.82, *p* = .166, *d* = 0.27. The difference between Chinese priming and the neutral priming was not significant, *t*(269) = 1.14, *p* = .488, *d* = 0.17.

The results were similar when controlling for all demographic variables. Culture priming significantly influenced dialectical self-concept, *F*(2,264) = 7.28, *p <* .001, *f*^2^ = .05.

**Figure S11**

*Priming American Culture Increased Dialectical Self-Concept for Mainland Chinese College Students*


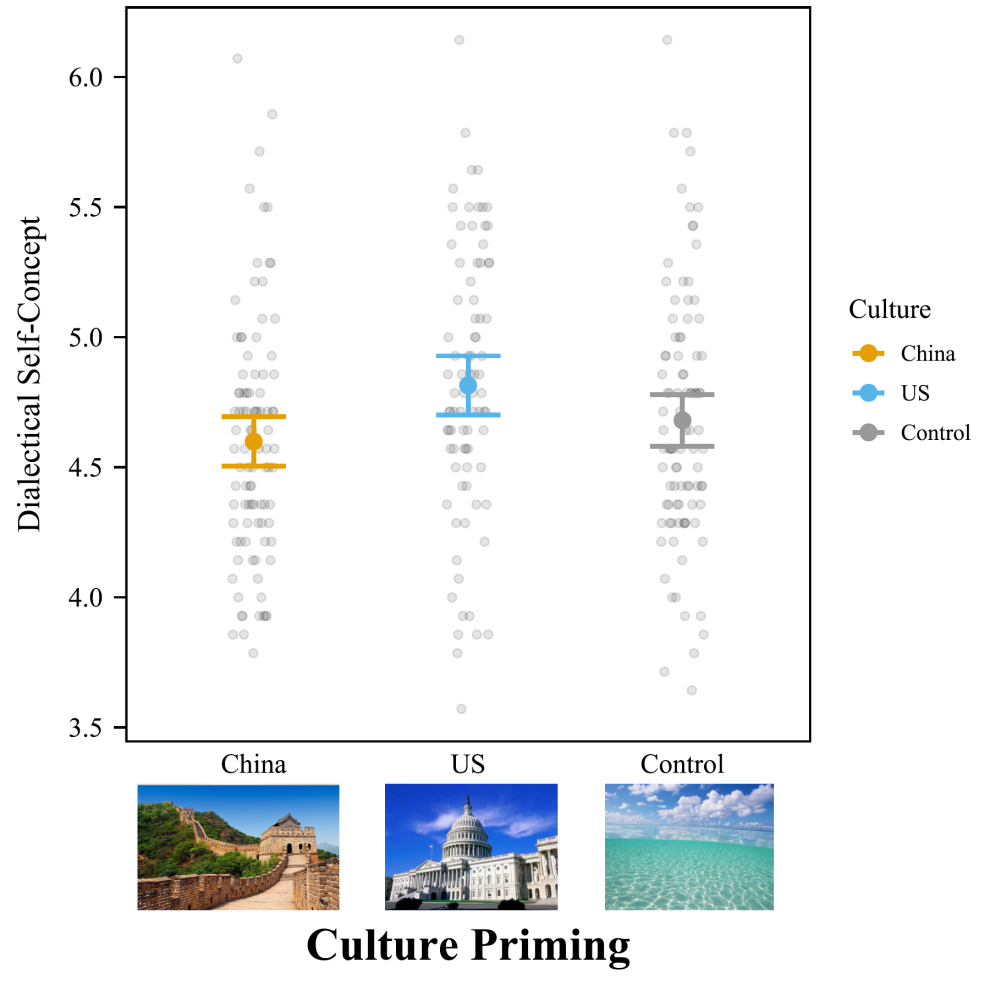


*Note:* Participants rated self-concept questions from 1 (*strongly disagree*) to 7 (*strongly agree*), such as “I sometimes believe two things that contradict each other.” Bars = 95% confidence intervals. Each dot represents a participant.

## Study 3:

Cultural priming did not affect older Chinese adults, *M_China_* = 4.23; *SD_China_* = 0.57; *M_America_* = 4.26; *SD_America_* = 0.48; *M_Control_* = 4.20; *SD_Control_* = 0.49, *F*(2,255) = 0.45, *p =* .637, *f^2^* = .004 (Figure S12). The results were similar when controlling for all demographic variables, *F*(2,251) = 0.28, *p* = .752, *f^2^* = .002.

**Figure S12**

*Cultural Priming Has No Effect on Attributions for Older Adults in Mainland China*


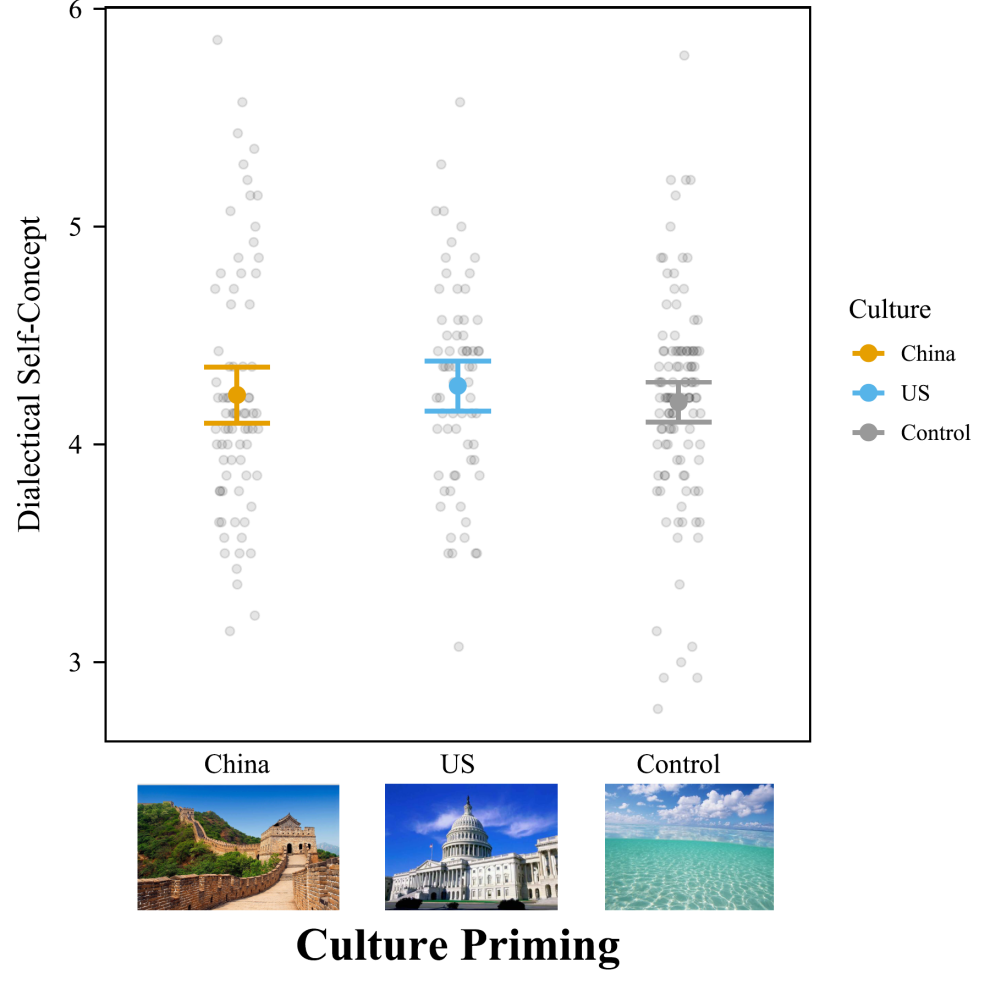


*Note:* Participants rated five attributions from 1 to 5. Participants in Study 3 were born before China’s Reform and Opening policy of 1978. Bars = 95% confidence intervals. Each dot represents a participant.

**Section 3: Results for the Full Sample (Without Excluding Participants)**

## Study 1

In the main text, we report analyses after excluding people who failed the attention check question. Here we report analyses including all participants. This helps test whether the results are robust to the decision to exclude participants based on the attention check question. The results are largely similar without excluding participants. This suggests that the conclusions do not depend on the decision to use the attention check question to exclude participants.

**Table S5**

*Correlations and Descriptive Statistics for Study 1 (N =120)*

| **Variable** | ***M*** | ***SD*** | **1** | **2** | **3** | **4** | **5** | **6** |
| --- | --- | --- | --- | --- | --- | --- | --- | --- |
| 1. Gender | 1.63 | 0.48 | — |  |  |  |  |  |
| 2. Age | 4.31 | 2.95 | –.08 | — |  |  |  |  |
| 3. Education | 2.10 | 0.51 | –.23^*^ | .46^***^ | — |  |  |  |
| 4. Experience Abroad (%) | 15.12% | 0.36 | .08 | .20^*^ | –.04 | — |  |  |
| 5. Liking for Pictures | 6.64 | 1.54 | –.03 | .13 | .05 | .04 | — |  |
| 6. Internal Attributions (Combine) | 3.01 | 0.41 | –.03 | –.09 | .02 | –.15 | .10 | — |
| 7. Dialectical Self-Concept (9-Item) | 4.30 | 0.51 | .14 | –.16 | –.15 | .10 | –.07 | –.04 |

*Note*. ^***^*p* < .001, ^**^*p* < .01, ^*^*p* < .05. Gender is coded 1 = male, 2 = female. Experience abroad is coded 0 = no, 1 = yes. Education is coded 1 = less than associate degree and equivalent, 2 = bachelor’s degree, 3 = master’s degree or higher. Liking is coded from 1 (*strongly dislike*) to 10 (*strongly like*).

### Attribution and Dialectical Self-Concept

A one-way ANOVA (1 = Chinese prime, 2 = American prime, 3 = Neutral prime) revealed a significant main effect of cultural priming on attribution (Table S6). Cultural priming significantly affected internal attributions, *F*(2,117) = 16.54, *p <* .001, *f^2^* = .282 [.129, .471] (Figure S13). Moreover, cultural priming also significantly affected dialectical self-concept, *F*(2,117) = 4.35, *p =* .015, *f^2^* = .074 [.008, .172](Figure S14). Participants with American priming had higher dialectical self-concept than control group (Table S6).

**Table S6**

*One-Way ANOVA on Attributions and Dialectical Self-Concept for Study 1*

|  | Culture Priming | | | *F* | *p* | Cohen’s *f*^2^ |
| --- | --- | --- | --- | --- | --- | --- |
|  | China | America | Control |  |  |  |
| Internal Attributions | 2.74±0.42^a^ | 3.20±0.36^b^ | 3.08±0.31^b^ | 16.54 | < .001 | .282 |
| Dialectical Self-Concept | 4.36±0.45^a^ | 4.43±0.61^b^ | 4.12±0.40^a^ | 4.35 | .015 | .074 |

*Note*: Distinct letters indicate statistically significant differences. Letters that are the same denote no significant difference.

**Figure S13**

*Participants Primed with American Culture Agreed More with Internal Attributions*

*
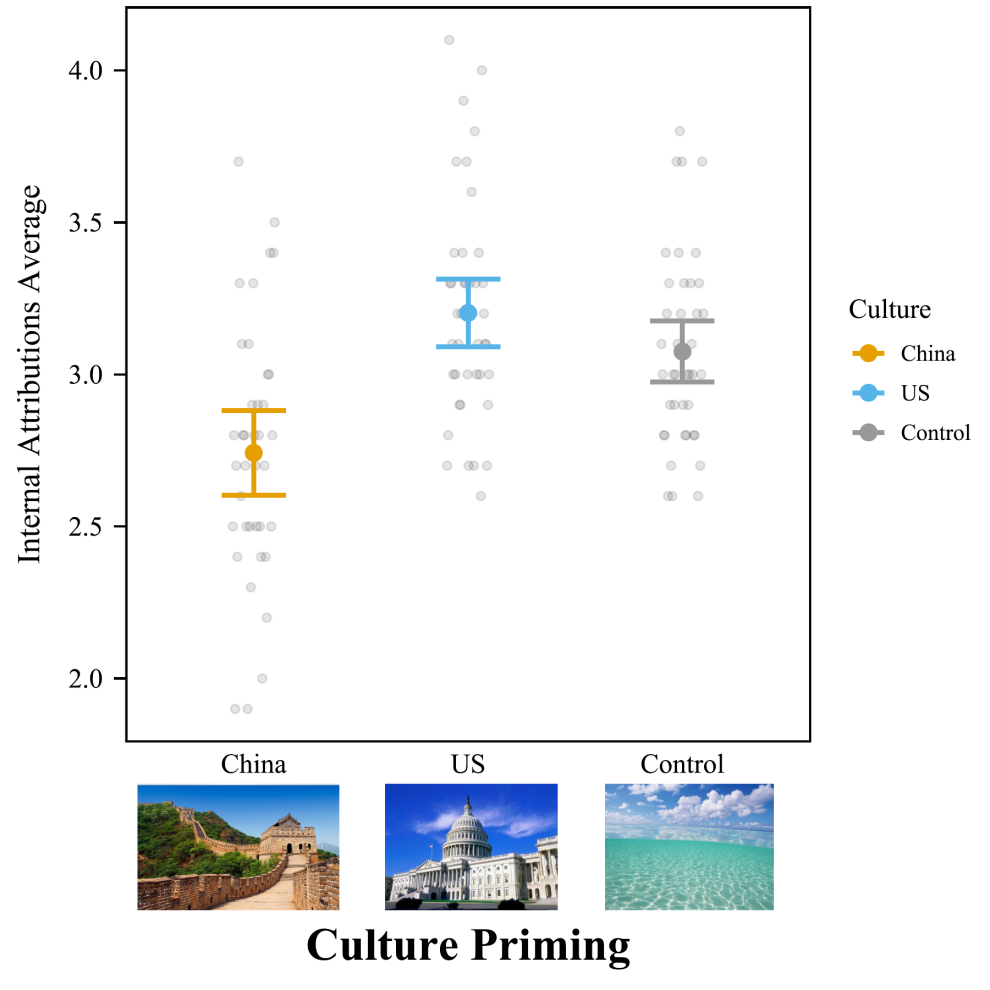
*

*Note*. The internal attribution score is calculated as the average of 10 questions, with participants providing ratings on internal attribution questions ranging from 1 to 5. The difference between American cultural priming and the control condition was not significant. Bars = 95% confidence intervals. Each dot represents a participant.

**Figure S14**

*American Culture Priming Increased Dialectical Self-Concept Over the Neutral Priming*

*
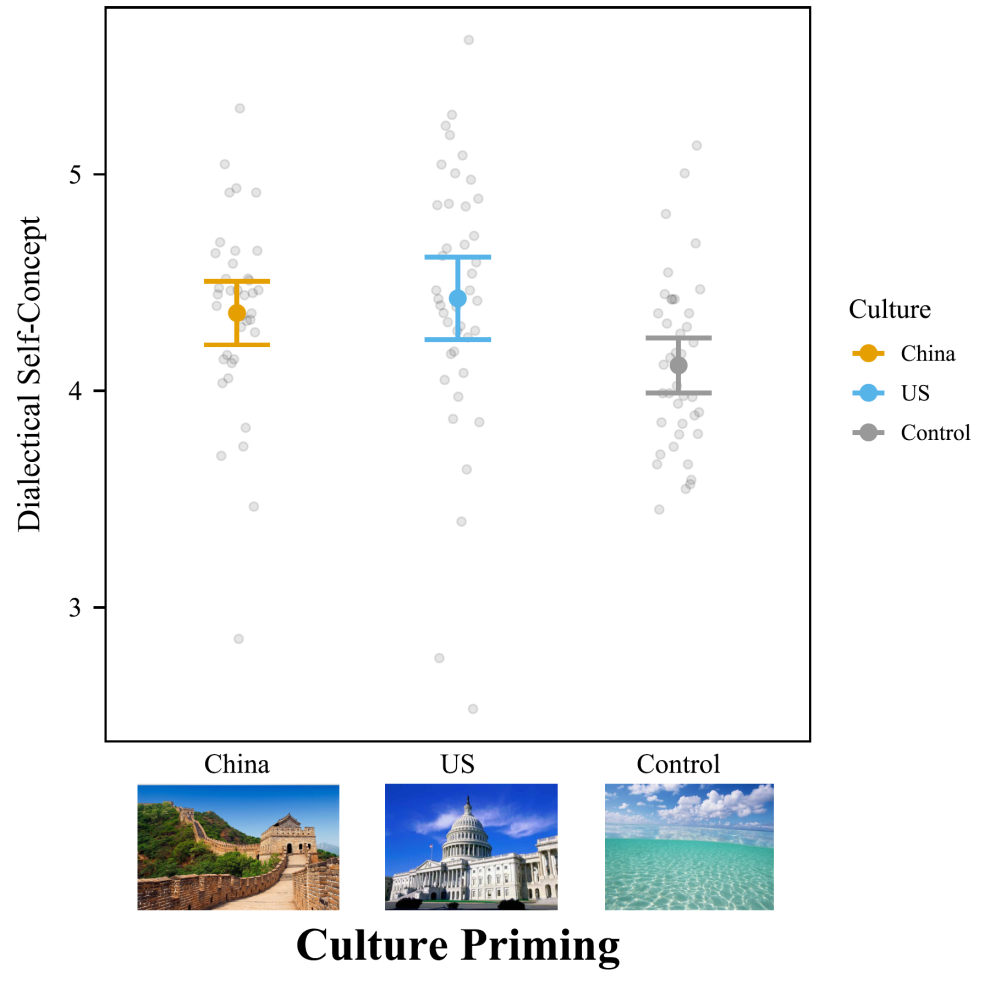
*

*Note:* Participants rated self-concept items from 1 (*strongly disagree*) to 7 (*strongly agree*), such as “I sometimes believe two things that contradict each other.” The difference between Chinese cultural priming and the control condition was not significant. Bars = 95% confidence intervals. Each dot represents a participant.

## Study 2

As with Study 1, we also re-ran the analyses with the full sample. In the main text, we used the attention check question to exclude participants who were not paying careful attention to the survey. Here we test whether the conclusions are robust to the decision to use the attention check by re-analyzing the data with all participants.

**Table S7**

*Correlations and Descriptive Statistics for Study 2 (N = 300)*

|  | *M* | *SD* | 1 | 2 | 3 | 4 | 5 | 6 |
| --- | --- | --- | --- | --- | --- | --- | --- | --- |
| 1. Gender | 1.65 | 0.48 | — |  |  |  |  |  |
| 2. Age | 22.72 | 4.07 | .02 | — |  |  |  |  |
| 3. Education | 1.30 | 0.61 | –.04 | .26^***^ | — |  |  |  |
| 4. Experience Abroad | 8.10% | 0.27 | .00 | .20^***^ | .37^***^ | — |  |  |
| 5. Liking of Pictures | 65.52 | 21.13 | –.03 | .00 | .00 | –.07 | — |  |
| 6. Internal Attributions | 3.16 | 0.38 | .03 | –.01 | –.08 | –.22^**^ | -.25^***^ | — |
| 7. Dialectical Self-Concept | 4.70 | 0.58 | –.06 | –.04 | .30^**^ | .05 | –.04 | .00 |

*Note*. ^***^*p* < .001, ^**^*p* < .01, ^*^*p* < .05. Gender is coded 1 = male, 2 = female. Experience abroad is coded 0 = no, 1 = yes. Education is coded 1 = less than high school and equivalent, 2 = vocational degree, 3 = bachelor’s degree, 4 = master’s degree, 5 = doctoral degree.

### Attribution and Dialectical Self-Concept

We conducted a one-way ANOVA on attributions across the three conditions (1 = Chinese prime, 2 = American prime, 3 = Neutral prime; Table S8). Cultural priming significantly affected internal attributions, *F*(2, 297) = 23.51, *p <* .001, *f^2^* = .159 [.086, .242] (Figure S15). Moreover, cultural priming marginal significantly affected dialectical self-concept, *F*(2,297) = 2.68, *p =* .070, *f^2^* = .018 [.000, .058](Figure S16).

**Table S8**

*ANOVA Testing the Effect of Cultural Priming on Attributions and Dialectical Self-Concept for Study 2*

|  | Culture Priming | | | *F* | *p* | Cohen’s *f*^2^ |
| --- | --- | --- | --- | --- | --- | --- |
|  | China | America | Control |  |  |  |
| Internal Attribution (Combined) | 2.98±0.36^a^ | 3.31±0.36^b^ | 3.22±0.35^b^ | 23.51 | < .001 | .159 |
| Dialectical Self-Concept | 4.63±0.52^b^ | 4.82±0.62^a^ | 4.67±0.59^ab^ | 2.68 | .070 | .018 |

*Note*: Distinct letters indicate statistically significant differences. Letters that are the same denote no significant difference.

**Figure S15**

*Priming American Culture Increased Internal Attributions for Mainland Chinese College Students*


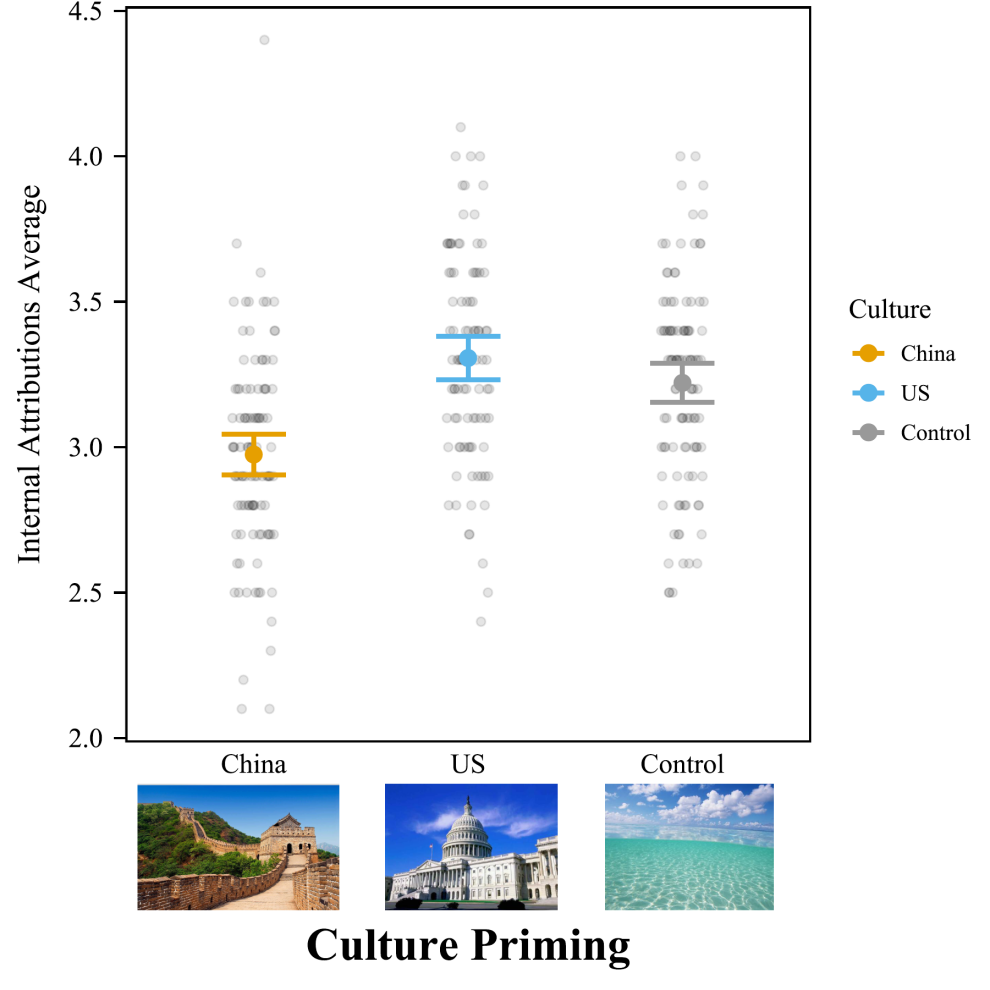


*Note:* Participants rated 10 internal attributions from 1 to 5. The difference between American cultural priming and the control condition was not significant. Bars = 95% confidence intervals. Each dot represents a participant.

**Figure S16**

*Priming American Culture Increased Dialectical Self-Concept for Chinese College Students*


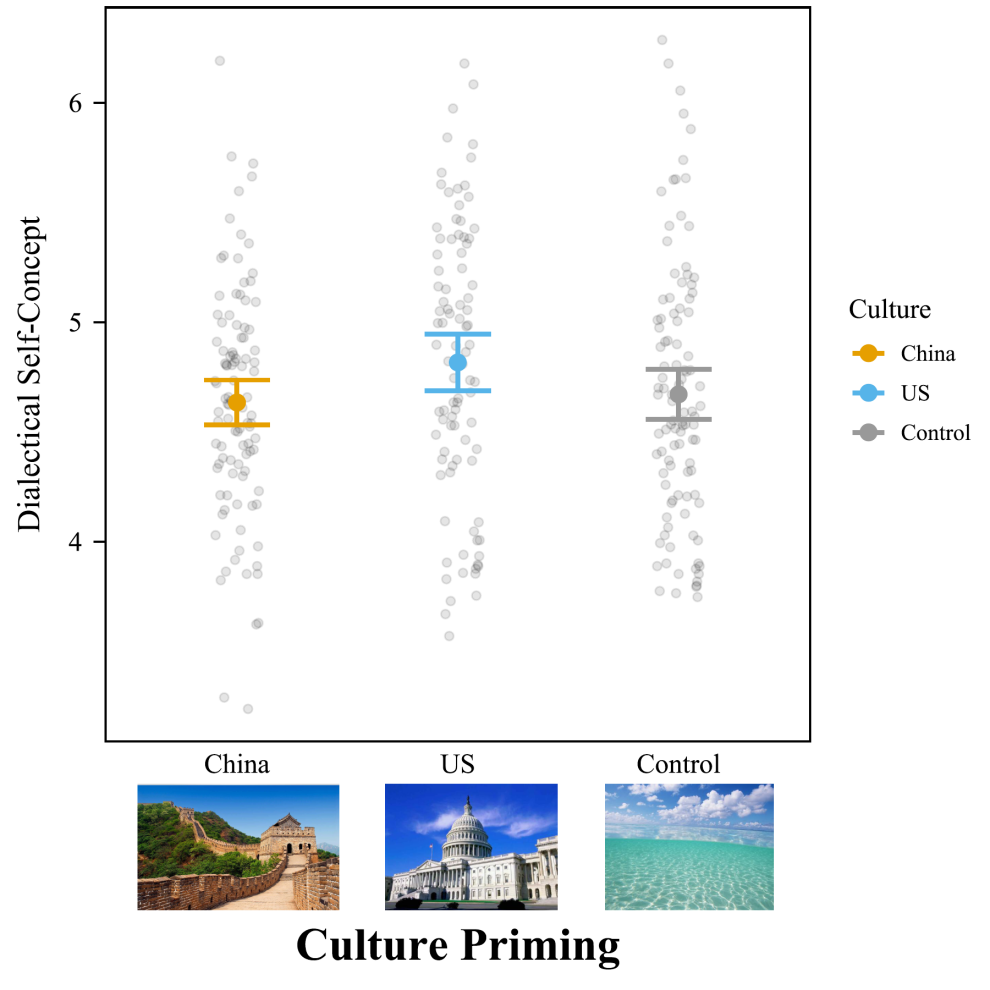


*Note:* Participants rated self-concept items from 1 (*strongly disagree*) to 7 (*strongly agree*). The difference between Chinese cultural priming and the control condition was not significant, nor was the American priming and the control condition. Bars = 95% confidence intervals. Each dot represents a participant.

## Study 3

In the main text, we excluded 42 older adults because they failed the attention check question (*n* = 9) or their age did not fit our criteria (*n* = 33). Here, we present analyses with all participants. This allows readers to check whether the conclusions are robust to the use of exclusion criteria.

**Table S9**

*Correlations and Descriptive Statistics for Study 3*

|  | *M* | *SD* | 1 | 2 | 3 | 4 | 5 | 6 |
| --- | --- | --- | --- | --- | --- | --- | --- | --- |
| 1. Gender | 1.44 | 0.50 | — |  |  |  |  |  |
| 2. Age | 52.81 | 9.24 | .06 | — |  |  |  |  |
| 3. Education | 2.61 | 1.26 | –.13^*^ | –.09 | — |  |  |  |
| 4. Experience Abroad | 0.05 | 0.21 | .03 | .00 | .14^*^ | — |  |  |
| 5. Liking of Pictures | 77.22% | 18.69 | –.01 | .02 | .01 | .01 | — |  |
| 6. Internal Attributions (Combined) | 2.94 | 0.43 | –.01 | .03 | .05 | .07 | –.05 | — |
| 7. Dialectical Self-Concept | 3.97 | 0.68 | .02 | .06 | –.03 | .00 | –.07 | .07 |

*Note*. ^***^*p* < .001, ^**^*p* < .01, ^*^*p* < .05. Gender is coded 1 = male, 2 = female. Experience abroad is coded 0 = no, 1 = yes. Education is coded 1 = less than high school and equivalent, 2 = vocational degree, 3 = bachelor’s degree, 4 = master’s degree, 5 = doctoral degree.

### Attribution and Dialectical Self-Concept

We conducted a one-way ANOVA on attributions across the three conditions (1 = Chinese prime, 2 = American prime, 3 = neutral prime; Table S10). Cultural priming did not affect internal attributions, *F*(2, 297) = 1.68, *p =* .189, *f^2^* = .011 [.000, .036] (Figure S17). But cultural priming did affect dialectical self-concept, *F*(2,297) = 6.13, *p =* .002, *f^2^* = .042 [.009, .086] (Figure S18). However, the pattern of the differences was hard to interpret. Participants primed with Chinese culture priming had higher dialectical self-concept than participants in the control group, *t*(297) = 3.49, *p* = .002, *d* = 0.469, 95% CI = [0.153, 0.786].

**Table S10**

*ANOVA Testing the Effect of Cultural Priming on Attributions and Dialectical Self-Concept for Study 3*

|  | Culture Priming | | | *F* | *p* | Cohen’s *f*^2^ |
| --- | --- | --- | --- | --- | --- | --- |
|  | China | America | Control |  |  |  |
| Internal Attributions (Combined) | 2.88±0.40^a^ | 3.00±0.45^a^ | 2.95±0.44^a^ | 1.68 | .189 | .011 |
| Dialectical Self-Concept | 4.15±0.72^b^ | 3.99±0.61^a^ | 3.83±0.67^a^ | 6.13 | .002 | .042 |

*Note*: Distinct letters indicate statistically significant differences. Letters that are the same denote no significant difference.

**Figure S17**

*Culture Priming Did Not Affect Internal Attributions for Older Adults Born Before Reform and Opening*


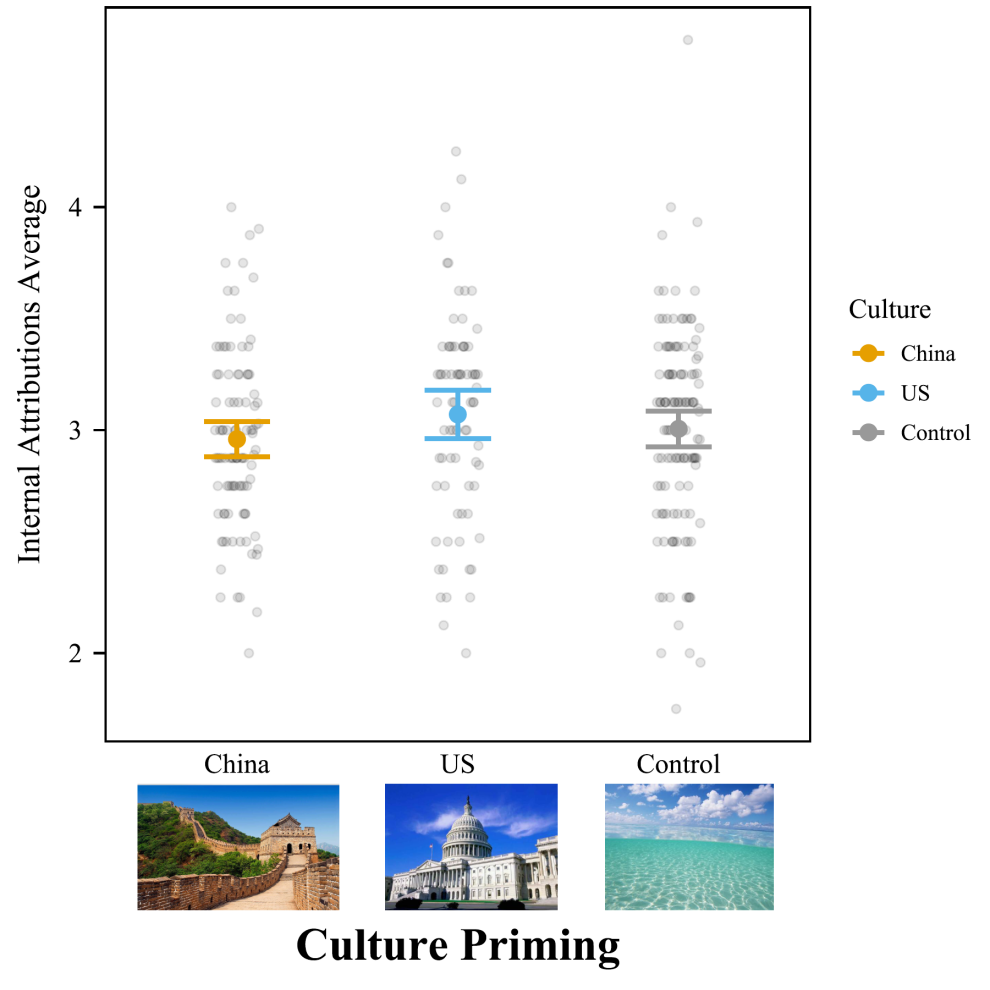


*Note:* Participants rated 10 internal attributions from 1 to 5. Bars = 95% confidence intervals. Each dot represents a participant.

**Figure S18**

*Priming Chinese Culture Increased Dialectical Self-Concept for Mainland Chinese Older Adults*


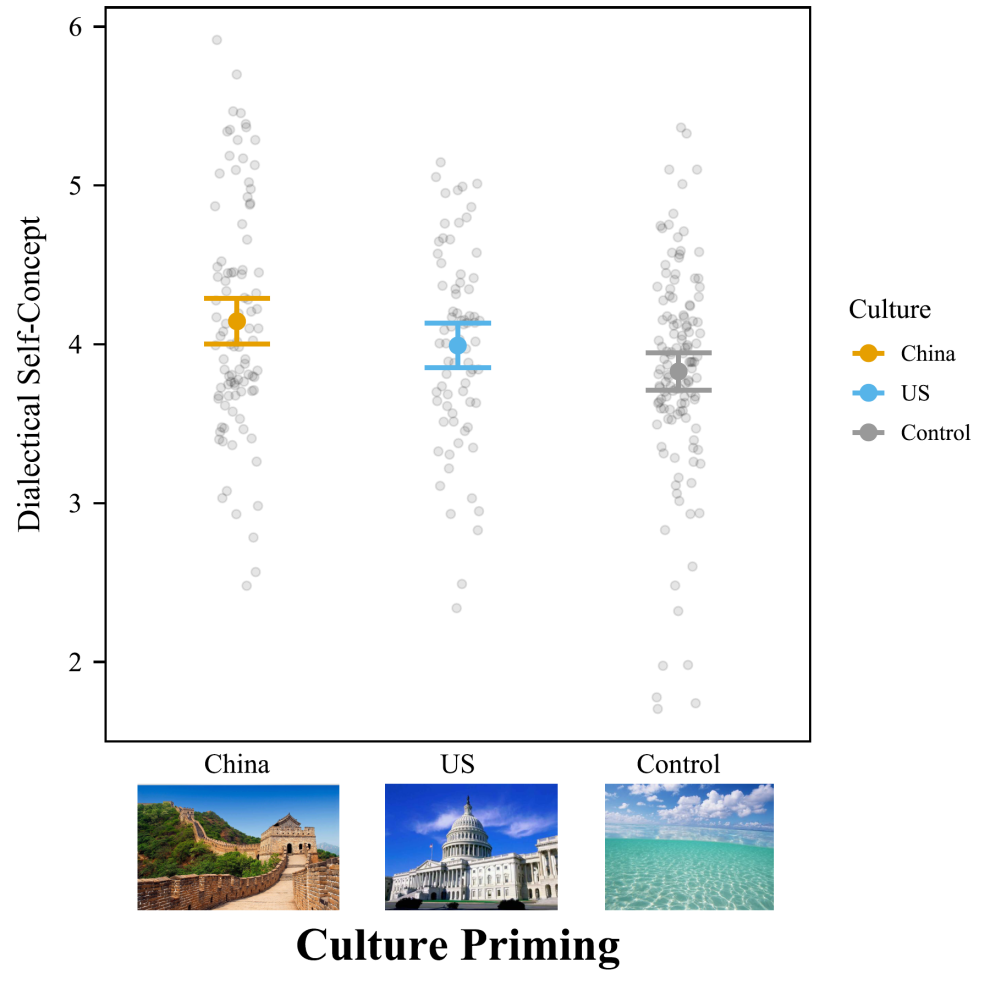


*Note:* Participants rated self-concept items from 1 (*strongly disagree*) to 7 (*strongly agree*), such as “I sometimes believe two things that contradict each other.” Bars = 95% confidence intervals. Each dot represents a participant.

# Section 4: Analysis Combining The Younger and Older Generations

We conducted 3 (Culture Priming: China, America, Control) × 2 (Generation: Younger vs. Older) ANOVAs with the full sample.

*Attribution:* The results showed that cultural priming significantly affected internal attributions, *F*(2,594) = 16.33, *p* < .001, *f^2^* = .055 [.026, .089]. We also ran ANOVAs controlling for all demographic variables, and the results were similar. Culture priming significantly influenced internal attributions, *F*(2,589) = 10.14, *p* < .001, *f^2^* = .034 [.012, .062]. Participants (Mainland students and the older generation) primed with American culture gave more internal attributions (*M* = 3.17; *SD* = 0.43) than people primed with Chinese culture (*M* = 2.93; *SD* = 0.38), *t*(597) = 5.61, *p* < .001, *d* = 0.587, 95% CI [0.341, 0.833], and people in the control condition (*M* = 3.07; *SD* = 0.42), *t*(597) = 2.32, *p* = .054, *d* = 0.235, 95% CI [-0.003, 0.473]. Additionally, participants in the control condition gave more internal attributions than students primed with Chinese culture, *t*(597) = 3.65, *p* < .001, *d* = 0.352, 95% CI [0.125, 0.579].

Age group also significantly affected internal attributions, *F*(1,594) = 48.81, *p* < .001, *f^2^* = .082 [.047, .126]. We also ran ANOVAs controlling for all demographic variables, and the results were similar. Culture priming significantly influenced internal attributions, *F*(1,589) = 12.12, *p* < .001, *f^2^* = .020 [.006, .045]. Undergraduate students gave more internal attributions (*M* = 3.16; *SD* = 0.38) than older participants (*M* = 2.94; *SD* = 0.43), *t*(598) = 6.85, *p* < .001, *d* = 0.560, 95% CI [0.399, 0.720].

The interaction effect was significant, *F*(2,594) = 4.12, *p* = .017, *f^2^* = .014 [.001, .032]. We also ran ANOVAs controlling for all demographic variables, and the results were similar, *F*(2,589) = 3.54, *p* = .030, *f^2^* = .014 [.012, .029]. Simple effects analyses found that college student with American culture priming had higher internal attributions, which was similar to ANOVA result (Table S11).

**Table S11**

*Simple Effect Analyses for Internal Attributions*

| Contrast | Difference | S.E. | *df* | *t* | *p* | Cohen’s *d* [95%CI] |
| --- | --- | --- | --- | --- | --- | --- |
| **Younger Generation** | | | | | | |
| America-China | 0.33 | 0.06 | 594 | 5.90 | < .001 | 0.847 [0.510, 1.183] |
| America-Control | 0.09 | 0.06 | 594 | 1.53 | .277 | 0.218 [-0.117, 0.553] |
| Control-China | 0.25 | 0.05 | 594 | 4.52 | < .001 | 0.628 [0.302, 0.955] |
| **Older Generation** | | | | | | |
| America-China | 0.12 | 0.06 | 594 | 1.95 | .125 | 0.299 [-0.061, 0.660] |
| America-Control | 0.05 | 0.06 | 594 | 0.84 | .678 | 0.122 [-0.220, 0.464] |
| Control-China | 0.07 | 0.05 | 594 | 1.32 | .386 | 0.177 [-0.139, 0.493] |

*Dialectical Self-Concept:* Cultural priming significantly affected dialectical self-concept, *F*(2,594) = 3.85, *p* = .022, *f^2^* = .013 [.001, .031]. We also ran ANOVAs controlling for all demographic variables, and the effect was significant, *F*(2,589) = 3.68, *p* = .026, *f^2^* = .012 [.001, .030]. Priming with American culture increased dialectical self-concept ratings (*M* = 4.45; *SD* = 0.74) over the neutral priming (*M* = 4.21; *SD* = 0.76, *t*[597] = 3.24, *p =* .004, *d* = 0.328, 95% CI = [0.090, 0.566]). Chinese cultural priming (*M* = 4.40; *SD* = 0.67) also led to higher dialectical self-concept than neutral priming, *t*(597) = 2.63, *p* = .024, *d* = 0.254, 95% CI [0.027, 0.480]. The difference between Chinese priming and the American priming was not significant, *t*(597) = 0.71, *p* = .756, *d* = 0.075, 95% CI [-0.171, 0.320].

Age group significantly affected dialectical self-concept, *F*(1,594) = 192.34, *p* < .001, *f^2^* = .245, 95% CI [.324, .412]. We also ran ANOVAs controlling for all demographic variables, and the results were similar, *F*(1,589) = 40.37, *p* < .001, *f^2^* = .068 [.037, .110]. Younger students have higher dialectical self-concept (*M* = 4.70; *SD* = 0.58) than older participants (*M* = 3.97; *SD* = 0.68), *t*(598) = 14.10, *p* < .001, *d* = 1.152, 95% CI [0.991, 1.312].

The interaction effect was significant, *F*(2,594) = 5.05, *p* = .007, *f^2^* = .017 [.003, .037]. We also ran ANOVAs controlling for all demographic variables, and the results were similar, *F*(2,589) = 5.69, *p* = .004, *f^2^* = .019 [.004, .041]. Simple effects analyses found that the college students primed with American culture had higher dialectical self-concept ratings, and older participants primed with Chinese culture had higher dialectical self-concept ratings (Table S12).

**Table S12**

*Simple Effect Analyses for Dialectical Self-Concept*

| Contrast | Difference | S.E. | *df* | *t* | *p* | Cohen’s *d* [95% CI] |
| --- | --- | --- | --- | --- | --- | --- |
| **Younger Generation** | | | | | | |
| America-China | 0.18 | 0.09 | 594 | 2.03 | .106 | 0.291[-0.046, 0.628] |
| America-Control | 0.15 | 0.09 | 594 | 1.63 | .235 | 0.232[-0.103, 0.566] |
| Control-China | 0.04 | 0.09 | 594 | 0.43 | .905 | 0.059[-0.267, 0.386] |
| **Older Generation** | | | | | | |
| America-China | -0.15 | 0.10 | 594 | -1.59 | .252 | 0.244[-0.604, 0.117] |
| America-Control | 0.16 | 0.09 | 594 | 1.78 | .176 | 0.260[-0.082, 0.602] |
| Control-China | -0.32 | 0.08 | 594 | -3.74 | <.001 | 0.503[0.187, 0.819] |

# Section 5: Analysis Combining The Hong Kong and Mainland Chinese Sample

We conducted 3 (Culture Priming: China, America, Control) × 2 (Sample: Hong Kong vs. Mainland) ANOVAs with full sample.

*Attribution:* The results showed that cultural priming significantly affected internal attributions, *F*(2,414) = 37.00, *p* < .001, *f^2^* = .179[.112, .255]. We also ran ANOVAs controlling for all demographic variables, and the results were similar. Culture priming significantly influenced internal attributions, *F*(2,409) = 28.96, *p* < .001, *f^2^* = .142[.083, .209]. Participants (Hong Kong and Mainland students) primed with American culture gave more internal attributions (*M* = 3.27; *SD* = 0.36) than people primed with Chinese culture (*M* = 2.91; *SD* = 0.39), *t*(417) = 8.25, *p* < .001, *d* = 0.995, 95% CI [0.712, 1.279], and people in the control condition (*M* = 3.18; *SD* = 0.36), *t*(417) = 2.14, *p* = .083, *d* = 0.256, 95% CI [-0.025, 0.538]. Additionally, participants in the control condition gave more internal attributions than students primed with Chinese culture, *t*(417) = 6.24, *p* < .001, *d* = 0.739, 95% CI [0.460, 1.018].

Sample group also significantly affected internal attributions, *F*(1,414) = 17.30, *p* < .001, *f^2^* = .042 [.015, .082]. We also ran ANOVAs controlling for all demographic variables, and the results were similar. Culture priming significantly influenced internal, *F*(1,409) = 12.25, *p* < .001, *f^2^* = .030 [.008, .065]. Mainland students gave more internal attributions (*M* = 3.16; *SD* = 0.38) than Hong Kong participants (*M* = 3.01; *SD* = 0.41), *t*(418) = 3.56, *p* < .001, *d* = 0.384, 95% CI [0.172, 0.597]. We speculate that innate biculturals and achieved biculturals may exhibit differing responses to cultural priming; however, direct evidence to test this hypothesis remains lacking. Therefore, more rigorous scientific research is necessary to provide empirical validation for this distinction.

The interaction effect was not significant, *F*(2,414) = 0.94, *p* = .390, *f^2^* = .005 [.000, .018]. We also ran ANOVAs controlling for all demographic variables, and the results were similar, *F*(2,409) = 2.14, *p* = .119, *f^2^* = .010 [.000, .031]. Simple effects analyses found that both Hong Kong and Mainland students with American culture priming had higher internal attributions, which was similar to ANOVA result (Table S13).

**Table S13**

*Simple Effect Analyses for Internal Attributions*

| Contrast | Difference | S.E. | *df* | *t* | *p* | Cohen’s *d* [95% CI] |
| --- | --- | --- | --- | --- | --- | --- |
| **Hong Kong Students** | | | | | | |
| America-China | 0.46 | 0.08 | 414 | 5.75 | < .001 | 1.287 [0.761, 1.814] |
| America-Control | 0.13 | 0.08 | 414 | 1.61 | .242 | 0.356 [-0.163, 0.876] |
| Control-China | 0.33 | 0.08 | 414 | 4.11 | < .001 | 0.931 [0.398, 1.464] |
| **Mainland Students** | | | | | | |
| America-China | 0.33 | 0.05 | 414 | 6.47 | < .001 | 0.928 [0.591, 1.265] |
| America-Control | 0.09 | 0.05 | 414 | 1.68 | .214 | 0.239 [-0.096, 0.574] |
| Control-China | 0.25 | 0.05 | 414 | 4.96 | < .001 | 0.689 [0.362, 1.016] |

*Dialectical Self-Concept:* Cultural priming significantly affected dialectical self-concept, *F*(2,414) = 4.86, *p* = .008, *f^2^* = .024 [.003, .052]. We also ran ANOVAs controlling for all demographic variables, and the effect was significant, *F*(2,409) = 6.98, *p* = .001, *f^2^* = .034 [.009, .068]. Priming with American culture increased dialectical self-concept ratings (*M* = 4.70; *SD* = 0.64) over the neutral priming (*M* = 4.52; *SD* = 0.60, *t*[417] = 2.52, *p =* .31, *d* = 0.302, 95% CI = [0.020, 0.583]). Chinese cultural priming (*M* = 4.56; *SD* = 0.52) led to similar dialectical self-concept to neutral priming, *t*(417) = 0.59, *p* = .825, *d* = 0.070, 95% CI [0.209, 0.349]. The difference between Chinese priming and the American priming was also not significant, *t*(417) = 1.92, *p* = .134, *d* = 0.232, 95% CI [-0.052, 0.515].

Sample group significantly affected dialectical self-concept, *F*(1,414) = 45.62, *p* < .001, *f^2^* = .110, 95% CI [.062, .172]. Mainland students have higher dialectical self-concept (*M* = 4.70; *SD* = 0.58) than Hong Kong participants (*M* = 4.30; *SD* = 0.51), *t*(418) = 6.61, *p* < .001, *d* = 0.714, 95% CI [0.502, 0.927]. While we also ran ANOVAs controlling for all demographic variables, and the results were not significant, *F*(1,409) = 0.29, *p* = .591, *f^2^* = .001 [.000, .011].

The interaction effect was not significant, *F*(2,414) = 1.81, *p* = .165, *f^2^* = .009 [.000, .027]. We also ran ANOVAs controlling for all demographic variables, and the result was significant, *F*(2,409) = 3.47, *p* = .032, *f^2^* = .017 [.001, .042]. Simple effects analyses found that both Hong Kong and Mainland students primed with American culture had higher dialectical self-concept ratings (Table S14).

**Table S14**

*Simple Effect Analyses for Dialectical Self-Concept*

| Contrast | Difference | S.E. | *df* | *t* | *p* | Cohen’s *d* [95% CI] |
| --- | --- | --- | --- | --- | --- | --- |
| **Hong Kong Students** | | | | | | |
| America-China | 0.07 | 0.13 | 414 | 0.54 | .850 | 0.122 [-0.405, 0.648] |
| America-Control | 0.31 | 0.12 | 414 | 2.52 | .032 | 0.557 [0.037, 1.076] |
| Control-China | 0.24 | 0.13 | 414 | -1.92 | .134 | 0.435 [-0.968, 0.098] |
| **Mainland Students** | | | | | | |
| America-China | 0.18 | 0.08 | 414 | 2.28 | .059 | 0.328 [-0.009, 0.665] |
| America-Control | 0.16 | 0.08 | 414 | 1.84 | .159 | 0.261 [-0.074, 0.596] |
| Control-China | 0.04 | 0.08 | 414 | 0.48 | .881 | 0.067 [-0.260, 0.394] |
